# Engineering PD-1-targeted small protein variants for in vitro diagnostics and in vivo PET imaging

**DOI:** 10.1186/s12967-024-05210-x

**Published:** 2024-05-06

**Authors:** Joanna Maria Mierzwicka, Hana Petroková, Leona Rašková Kafková, Petr Kosztyu, Jiří Černý, Milan Kuchař, Miloš Petřík, Kateřina Bendová, Kristýna Krasulová, Yaroslava Groza, Lucie Vaňková, Shiv Bharadwaj, Natalya Panova, Michal Křupka, Jozef Škarda, Milan Raška, Petr Malý

**Affiliations:** 1 Laboratory of Ligand Engineering, Institute of Biotechnology of the Czech Academy of Sciences, BIOCEV Research Center, Průmyslová 595, 252 50 Vestec, Czech Republic; 2 Department of Immunology, University Hospital Olomouc, Zdravotníků 248/7, 77900 Olomouc, Czech Republic; 3 Department of Immunology, Faculty of Medicine and Dentistry, Palacky University Olomouc, Hněvotínská 3, 779 00 Olomouc, Czech Republic; 4 Laboratory of Structural Bioinformatics of Proteins, Institute of Biotechnology of the Czech Academy of Sciences, BIOCEV Research Center, Průmyslová 595, 252 50 Vestec, Czech Republic; 5 Institute of Molecular and Translational Medicine, Faculty of Medicine and Dentistry and Czech Advanced Technology and Research Institute, Palacky University Olomouc, Hněvotínská 5, 779 00 Olomouc, Czech Republic; 6 Institute of Clinical and Molecular Pathology, Faculty of Medicine and Dentistry, Palacky University Olomouc, Hněvotínská 3, 779 00 Olomouc, Czech Republic

**Keywords:** Immune checkpoint, Programmed cell death 1, Non-small cell lung cancer, Cancer diagnostic, Combinatorial library, Protein engineering

## Abstract

**Background:**

Programmed cell death 1 (PD-1) belongs to immune checkpoint proteins ensuring negative regulation of the immune response. In non-small cell lung cancer (NSCLC), the sensitivity to treatment with anti-PD-1 therapeutics, and its efficacy, mostly correlated with the increase of tumor infiltrating PD-1^+^ lymphocytes. Due to solid tumor heterogeneity of PD-1^+^ populations, novel low molecular weight anti-PD-1 high-affinity diagnostic probes can increase the reliability of expression profiling of PD-1^+^ tumor infiltrating lymphocytes (TILs) in tumor tissue biopsies and in vivo mapping efficiency using immune-PET imaging.

**Methods:**

We designed a 13 kDa β-sheet Myomedin scaffold combinatorial library by randomization of 12 mutable residues, and in combination with ribosome display, we identified anti-PD-1 Myomedin variants (MBA ligands) that specifically bound to human and murine PD-1-transfected HEK293T cells and human SUP-T1 cells spontaneously overexpressing cell surface PD-1.

**Results:**

Binding affinity to cell-surface expressed human and murine PD-1 on transfected HEK293T cells was measured by fluorescence with LigandTracer and resulted in the selection of most promising variants MBA066 (hPD-1 KD = 6.9 nM; mPD-1 KD = 40.5 nM), MBA197 (hPD-1 KD = 29.7 nM; mPD-1 KD = 21.4 nM) and MBA414 (hPD-1 KD = 8.6 nM; mPD-1 KD = 2.4 nM). The potential of MBA proteins for imaging of PD-1^+^ populations in vivo was demonstrated using deferoxamine-conjugated MBA labeled with ^68^Galium isotope. Radiochemical purity of ^68^Ga-MBA proteins reached values 94.7–99.3% and in vitro stability in human serum after 120 min was in the range 94.6–98.2%. The distribution of ^68^Ga-MBA proteins in mice was monitored using whole-body positron emission tomography combined with computerized tomography (PET/CT) imaging up to 90 min post-injection and *post mortem* examined in 12 mouse organs. The specificity of MBA proteins was proven by co-staining frozen sections of human tonsils and NSCLC tissue biopsies with anti-PD-1 antibody, and demonstrated their potential for mapping PD-1^+^ populations in solid tumors.

**Conclusions:**

Using directed evolution, we developed a unique set of small binding proteins that can improve PD-1 diagnostics in vitro as well as in vivo using PET/CT imaging.

**Graphical Abstract:**

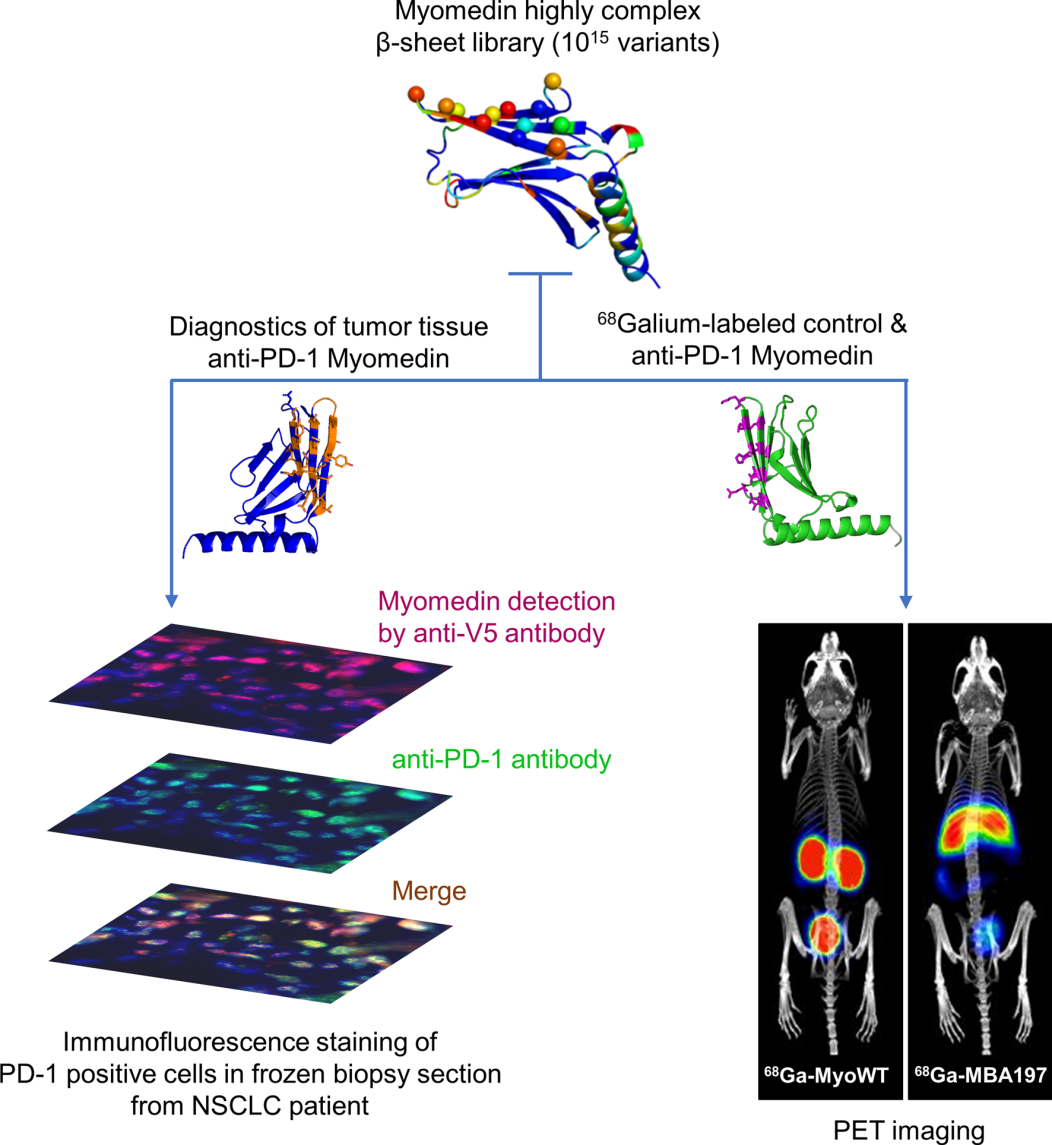

**Supplementary Information:**

The online version contains supplementary material available at 10.1186/s12967-024-05210-x.

## Introduction

Immune checkpoint molecules are cellular receptors that modulate the immunosuppressive signaling pathways. These molecules are essential for maintaining autoimmune reactions and dampening the excessive activation of immune cells following combating the infection or other threats to minimize the collateral damages [1]. However, overexpression or overactivation of immune checkpoint receptors can cause inhibition or exhaustion of effector immune cells, especially T cells [2]. It is now clear that many cancers enhance immunosuppressive interactions of local immune cells to avoid immune surveillance and clearance, thus promoting cancer growth [1]. Accordingly, growing efforts to target these inhibitory receptors for gaining anti-cancer immunity have led to the clinical development and licensing of immune checkpoint inhibitors (ICIs) as the therapy for many types of cancer [3, 4].

PD-1 (B7-H1; also CD279) receptor is expressed on the surface of activated B cells, T cells, monocytes, natural killer cells, some myeloid cells and cancer cells [5, 6]. Under normal physiological conditions, upon docking with its ligands (PD-L1 and PD-L2), PD-1 on immune cells promotes a signaling cascade in maintaining the balance between self-tolerance and reactivity to extraneous signals [7]. However, in the context of malignancy, tumor and stromal PD-L1 expression poses a barrier to immune function by promoting the exhaustion of the antitumor T cell population that might otherwise contribute to tumor cells elimination [8]. For instance, high levels of PD-1 expression are detected during some chronic infections, and in exhausted tumor-infiltrating CD8^+^ cells in many types of cancer [9]. Consequently, the PD-1/PD-L1 pathway has emerged as a critical target for monoclonal antibodies-based inhibitors development targeting PD-1/PD-L1 interaction. Such ICIs have demonstrated impressive activity against many cancers [10–15].

Recent clinical trials exploring the use of ICIs, including monoclonal antibodies, as antagonists of PD-1 or PD-L1 receptors have shown significant improvement in the survival rate of patients with advanced non-small-cell lung cancer (NSCLC) [16, 17]. However, the responses of cancer patients to ICIs vary in success and approximately 60% to 70% of patients’ progress within 6 months after ICI therapy initiation [11, 18, 19]. In this context, a recent study illustrated that assessing the immune profile of tumor biopsies in the early course of ICI therapy was a better predictor of response than assessing pretreatment samples [20]. Thereof, unmet needs exist in predicting such responses with accurate biomarkers to maximize the efficacy and minimize the toxicity of ICIs, in particular, to identify patients that are less likely to benefit from ICI therapy [20].

In analogy to molecular biomarkers that have been used for the identification of patients with targetable oncogenes [21], it has been assumed that tumor-infiltrating T lymphocytes (TILs) as predictive biomarker for clinical benefit to PD-1 blockade in patients with advanced NSCLC [22–24]. In this context, pembrolizumab and nivolumab as anti-PD-1 checkpoint inhibitors have been reported to predict the prognostic significance of TILs in a variety of solid tumors, including NSCLC [24, 25]. However, antibodies have some drawbacks due to their large size, including slower clearance from the system [26] and lower penetrance into the solid tumors [27]. For example, PD-1–expressing effector T cells are found infiltrated within solid tissue of PD-L1–expressing tumors [8]. Therefore, antibodies may fail to exhibit penetration and substantial binding to PD-1–expressing T cells within solid tumors, such as NSCLC, which may be leading to suboptimal prognostic score.

For a high efficacy and clinical response to ICI therapy, mapping of PD-1 expression on tumor-infiltrating immune cells and scoring the tumor infiltration but also PD-1 expression on tumor cells is necessary before a treatment strategy decision is made. However, heterogeneity of tumor tissue infiltration and space-and-time changes in PD-1 expression are limiting factors for the easy and reliable detection and scoring of PD-1^+^ TILs [28, 29]. In addition, some detection antibodies are susceptible to a variable quality and a loss of specificity or high binding affinity [27, 30, 31]. Therefore, the development of small non-toxic agents with an increased tissue penetration is of general interest.

In this work, we used directed evolution by ribosome display to generate a collection of PD-1-targeted small protein variants of MBA series that were derived from 13 kDa Myomedin scaffold library using randomization of 12 mutable residues located on a flat β-sheet surface. We provide evidence that MBA proteins exhibit high affinity and specificity to human and mouse recombinant or cell surface-exposed PD-1 receptors and can be used as non-immunoglobulin alternatives for monitoring PD-1^+^ cell populations in tumor tissue biopsy samples as well as for imaging of organs/tissues containing PD-1^+^ cells in vivo. Thus, these engineered MBA proteins can serve as beneficial diagnostic tools for the clinical management of NSCLC patients.

## Materials and methods

### Myomedin combinatorial library design

**Fig. 1:**
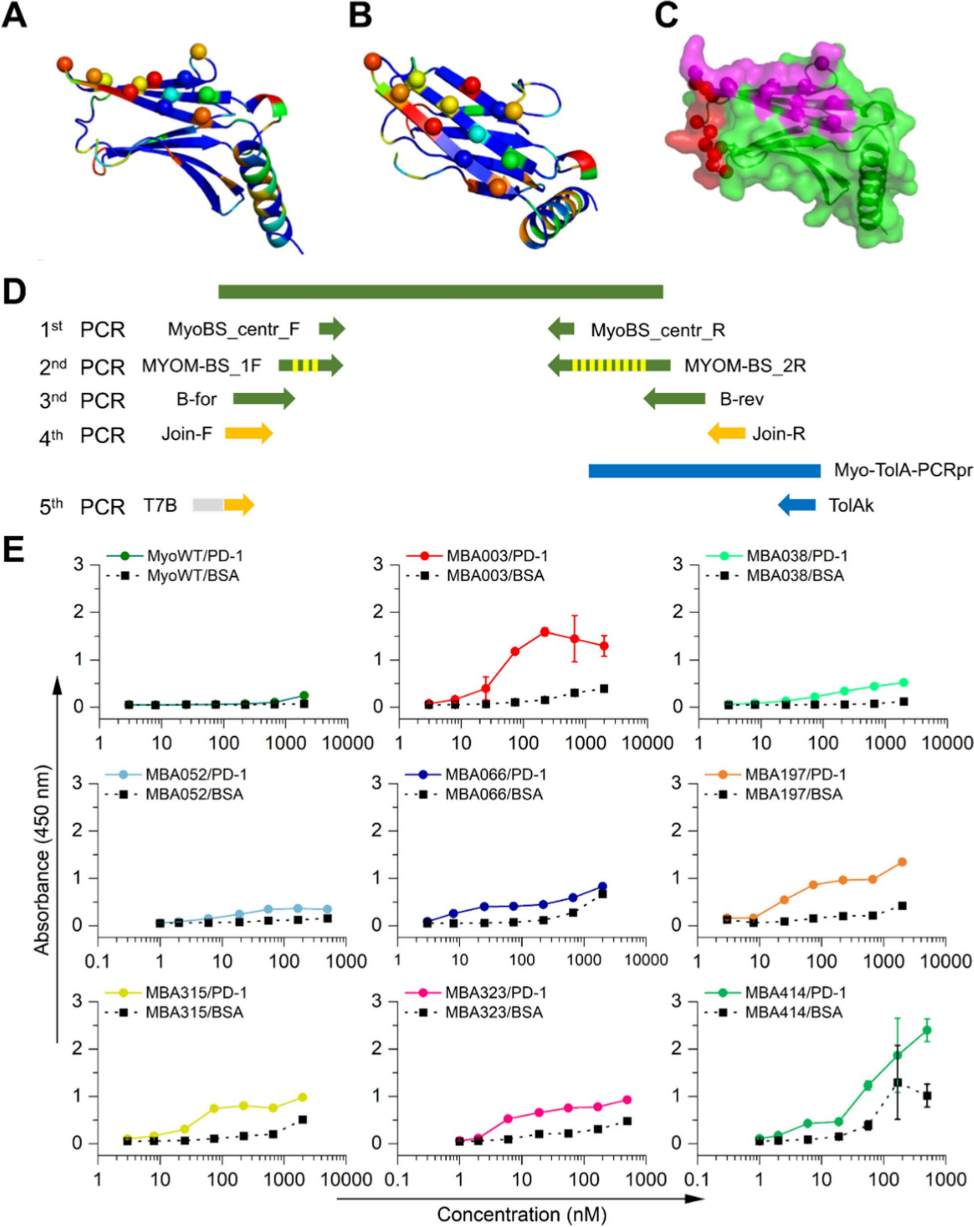
Concept of Myomedin β-sheet combinatorial library and selected MBA variants’ binding curves measured by ELISA. **A** Side view and **B** top view to the human myomesin-1 domain 10 in cartoon representation, showing positions of residues selected for randomization. Highlighted are the C_α_ positions of residues N1276, I1278, E1281, T1315, T1317, Q1319, Q1321, K1324, T1326, H1328, T1330, and V1332 numbered according to the UniProt (UniProt, 2019) P52179 record. The residue color scale represents the mutability score, from blue to red for low to high mutability. **C** Surface representation of the Myomedin scaffold with the β-sheet patch in magenta and the previously defined Myomedin loop library in red [34]. **D** Scheme of Myomedin β-sheet library assembly using multistep PCR. **E** Binding of selected MBA variants to recombinant hPD-1 receptor assessed by ELISA assay. Detection of the signal was performed using anti-V5 tag-HRP conjugated antibody at 450 nm

The β-sheet Myomedin combinatorial library was derived from structure of human myomesin-1 domain 10. The residues suitable for randomization were identified by in silico mutability screening of all amino acid residues resolved in the crystal structure of human myomesin domains 10 and 11 (PDB ID 3rbs) [32]. All residues were mutated to all 20 amino acids using the PositionScan routine of FoldX program [33] and a mutability score corresponding to a number of stabilizing substitutions was assigned to each residue. The mutability score was also calculated for the previously characterized stable isolated myomesin-1 domain 10 structure (PDB ID 6t3o) (Fig. 1A–C) [34].

### Construction and assembly of Myomedin β-sheet library

A series of PCR steps was used for the construction of Myomedin β-sheet combinatorial library using Q5® High-Fidelity DNA Polymerase (NEB) (Fig. 1D) and a set of primers listed in Additional file 1: Table S1. Individual PCR products were separated in 1–2% agarose and DNA of the desired length was isolated using gel extraction kit (Monarch, NEB). In the 1st PCR of internal non-randomized part of the library was amplified with primers MyoBS_centr _F, MyoBS_centr _R and template from Myomedin wild-type (MyoWT) (64 °C melting temperature, 30 cycles) giving 87 bp product. In the 2nd PCR randomized parts of the library were attached with primers MYOM-BS_1F and MYOM-BS_2R (64 °C melting temperature, 30 cycles) resulting in 210 bp product. By 3rd PCR Myomedin scaffold was completed with primers B-for and B-rev (65 °C melting temperature, 30 cycles) with the product of 333 bp. For use in ribosomal display, RBS, TolA spacer and stem-loops were attached to the library scaffold in two more PCR reactions, as described earlier [34].

### Ribosome display

Three rounds of ribosome display selection were performed as described earlier [34] with the following differences. Human PD-1 (hPD-1) protein (R&D Systems) was coated into the wells of MaxiSorp 96-well plate (NUNC) for 1 h at room temperature (RT) with the concentration 2 µg/ml in the 1st and 2nd round and 0.5 µg/ml in the 3rd round. Preselection step was performed in wells just blocked by 3% BSA. Washing of complex of ribosomes RNA and protein attached to the hPD-1 in the well of microtiter plate was done with increasing stringency: 5 times (10 times and 10 times) with TBS-T (50 mM Tris, 150 mM NaCl pH 7.5 with 0.05%, 0.05% and 0.2% of Tween-20, in 1st, 2nd and 3rd round respectively). After 3rd round of selection, resulting cDNA was cloned into the vector pET28b and vector was inserted into the *E. coli* XL1-blue host cells. Randomly selected colonies were sequenced and those with the mutations in randomized positions only were cloned into *E. coli* BL21 (DE3) cells and screened for binding to the hPD-1 in ELISA assay. Construct of MBA variants contains N-terminal His-tag used for purification and C-terminal V5-tag used for specific detection.

### In silico modeling by docking

The MBA variants were modeled using the MODELLER 9v14 suite of programs [35] based on the non-mutated myomesin-1 domain 10 structure (PDB ID 3rbs) [32], residues 1246-1358). The model of the extracellular domain of hPD-1 (residues 24 to 170 according to the Q15116 UniProt record) and the hPD-1/hPD-L1 complex were built using local installation of AlphaFold [36]. Flexible side chain protein–protein global docking was performed using a local copy of the ClusPro server [37, 38] docking the MBA variants (as ligands) to the hPD-1 model (as the receptor). The docking results were analyzed using PyMOL version 2.6.0 (The PyMOL Molecular Graphics System, Schrödinger, LLC.)

### Bioconjugation of MBA binders

Bioconjugation of MBA proteins was performed using *p*-isothiocyanatobenzyl-deferoxamine (p-NCS-Bz-DFO; CheMatech, France). Briefly, 0.5 mg of particular binder in elution buffer was dialyzed into 0.5 ml of PBS. The pH was adjusted to 9 with 0.1 M Na_2_CO_3_ and p-NCS-Bz-DFO diluted in DMSO was added in 3 molar excess. Mixture was incubated for 1 h at 37 °C. Unreacted deferoxamine was removed and buffer exchange into water was performed using Zeba spin desalting column (Thermo Fisher Scientific, MA, USA).

### ^68^Galium-labeling of MBA proteins

DFO-conjugated binders were radiolabeled with ^68^Galium (^68^Ga) 100 µl of MBA binders (0.1–1.4 mg/ml) were mixed with 30 μl of sodium acetate (155 mg/ml) and 300 μl (25–50 MBq) of ^68^Ga-eluate from ^68^Ge/^68^Ga-generator (Eckert and Ziegler). The mixture was incubated for 15 min at room temperature. Radiochemical purity was then assessed by iTLC chromatography using two different mobile phases, A) 0.1 M sodium citrate and B) 1 M sodium acetate + methanol (1:1).

### In vivo and ex vivo analysis of biodistribution of MBA proteins in mouse tissues

All the animal experiments were conducted in accordance with regulations and guidelines of the Czech Animal Protection Act (No. 246/1992), and with the approval of the Czech Ministry of Education, Youth, and Sports (MSMT-24421/2021-4) and the institutional Animal Welfare Committee of the Faculty of Medicine and Dentistry of Palacky University in Olomouc. 10 weeks old female Balb/c mice were housed under standard laboratory conditions. The number of mice used in biodistribution study was reduced as much as possible to three mice for each binder. The labeled binders for ex vivo biodistribution were retro-orbitally injected into mice at the dose of 100 μl representing 1–2 MBq of ^68^Ga and 1.5–3 μg of the peptide. The animals were sacrificed 30- and 90-min post-injection by cervical dislocation. The blood, spleen, pancreas, stomach, intestines, kidneys, liver, heart, lungs, muscle, and bone were collected and weighed. The radioactivity of obtained tissue samples was measured in a γ-counter (2480 Wizard^2^ automatic gamma counter; PerkinElmer, Waltham, USA). The biodistribution data were calculated as the percentage of injected dose per gram of tissue (% ID/g).

Experimental animals for PET/CT imaging were retro-orbitally injected with ^68^Ga labelled MBA binders (approximately ∼ 4–6 μg of binder) at a dose of 6–8 MBq per animal and placed in the PET/CT scanner (nanoScan PET/CT, Mediso Medical Imaging Systems, Budapest, Hungary) immediately after the injection of the binder for dynamic imaging or 45 min after the injection for static imaging. A 10 min PET scan was performed for static imaging, while dynamic PET imaging was performed for 90 min in the form of 18 consecutive 5 min PET scans allowing monitoring of temporal changes in connective tissue biodistribution, followed by whole-body helical CT scan (50 kVp/980 μA, 720 projections).

### Mouse infection model

In vivo distribution of ^68^Ga-MBA066 and ^68^Ga-MyoWT was studied in a mouse model of acute myositis. The murine myositis model was established by intramuscular injection of *E. coli* CCM 5172 suspension. Mice were inoculated with 5 × 10^7^ CFUs of live bacteria in the left hind leg and saline in the right hind leg. The microbial infection was allowed to develop for 5 h. The animals were then injected with ^68^Ga-labeled binders and imaged by PET/CT as described above.

### Immunohistochemistry staining

Tissues were collected into OCT medium and immediately frozen on dry ice and transferred into − 80 °C where stored until analysis. Sections were cut on cryotome machine, hydrated with PBS, fixed in 4% formaldehyde, and blocked with PBS + 1% FBS for 1 h. 100 ng of MBA414 or 1 µg of anti-PD-1 antibody-FITC (Novus) were left overnight at 4 °C. Anti-V5 Alexa Fluor 647 antibody (Invitrogen) was used next day for 1 h. Mounting medium with DAPI (Merck) was used for final preparation of specimen for microscopy. Microscope Leica connected with camera was used for observation of tissues, software was used for preparation of images. Co-localization was quantified by ImageJ software (LOCI, University of Wisconsin) supplemented with plugin JACoP (Fabrice P. Cordelières, Bordeaux Imaging Center (France). Fabrice.Cordelieres@gmail.com, Susanne Bolte, IFR 83, Paris (France). Susanne.Bolte@upmc.fr).

Additional part of methodology is provided in Additional file. In this study, selection criteria for experimental analysis of MBA variants is given as Additional file 1: Table S2.

## Results

### Concept and description of Myomedin β-sheet combinatorial library

In our previous work, we demonstrated that the human myomesin-1 domain 10 structure exhibited sufficient stability to be used as a scaffold for the generation of a highly complex combinatorial library using randomization of 12 mutable amino acid residues located in the three-domain loops, named Myomedin scaffold [34, 39]. Based on the available crystal structure of mouse/human PD-1/PD-L1 complex (PDB ID 3bik), it seems to be evident that critical interaction residues of human PD-L1 required for the recognition of PD-1 receptor are located on β-sheet surface (Additional file 1: Fig. S1a). Due to the substantial structural similarity between human PD-L1 and Myomedin scaffold (see the superposition in Fig. S1b and built human/human PD-1/PD-L1 complex homology model in Additional file 1: Fig. S1c), we first investigated a mutability potential for amino acid residues within the cognate Myomedin β-sheets.

The mutability score was used as a criterion for the final selection of candidate residues to find a continuous patch of accessible surface residues with high mutability (suggesting a lower probability of overall fold destabilization upon randomization). Herein, an in silico procedure identified a patch of suitable residues localized on the β-sheet surface of human myomesin-1 domain 10 (Fig. 1A–C). The residues N1276, I1278, E1281, T1315, T1317, Q1319, Q1321, K1324, T1326, H1328, T1330, and V1332 numbered according to the UniProt [40] P52179 record were used for randomization. The selected β-sheet residue patch (Fig. 1C magenta) is located in the proximity of loop-randomized surface sharing a single amino acid residue (Fig. 1C red). The theoretical complexity of the designed Myomedin β-sheet library is 2 × 10^15^ variants.

### Myomedin combinatorial library assembly and identification of PD-1 binding proteins

The new type of highly complex Myomedin combinatorial library carrying 12 mutable amino acid residues located in the β-sheet region was engineered using a set of primers (Additional file 1: Table S1) in multiple PCR reactions (Fig. 1D). Randomization of amino acid codons within the primers was introduced by synthetic trinucleotide technology (TRIM) with the elimination of cysteine residue to prevent the formation of disulfide bridges. After assembly of full-length DNA library and its insertion into a plasmid cloning vector, *E. coli* XL1 cells were transformed with the generated cDNA plasmid library and randomly picked bacterial colonies were used to sequence individual DNA samples for the evaluation of the quality of DNA library and verification of a random codon distribution in the predesigned mutable residues.

To select binders specific to hPD-1 from the Myomedin β-sheet library, three rounds of ribosome display were performed with recombinant hPD-1 receptor as a molecular target immobilized in a 96-well microtiter plate. Then, the library of DNA transcripts was inserted into the plasmid vector by molecular cloning and the generated cDNA plasmid library was used to transform *E. coli* XL1-Blue cells. A total of 378 individual colonies were screened by colony PCR and 220 variants with expected PCR product size were further sequenced. Of them, 75 variants with required mutations solely in the randomized regions were used to transform *E. coli* BL21 (DE3) cells and bacterial lysates of these Myomedin-containing clones were tested in ELISA assay for binding to immobilized hPD-1. A total of 25 MBA variants with specific binding to PD-1 were produced and purified on Ni–NTA agarose matrix and particular binding curves to hPD-1 were analyzed by ELISA. Finally, Myomedin variants MBA003, MBA038, MBA052, MBA066, MBA197, MBA315, MBA323, and MBA414 binding to recombinant hPD-1 (Fig. 1E) were selected for further characterization. Variants MBA038 and MBA066 were produced in cytosolic extract while variants MBA003, MBA052, MBA197, MBA315, MBA323, and MBA414 were present in inclusion bodies.

### MBA variants exhibit specific binding to cell-surface expressed human PD-1

**Fig. 2:**
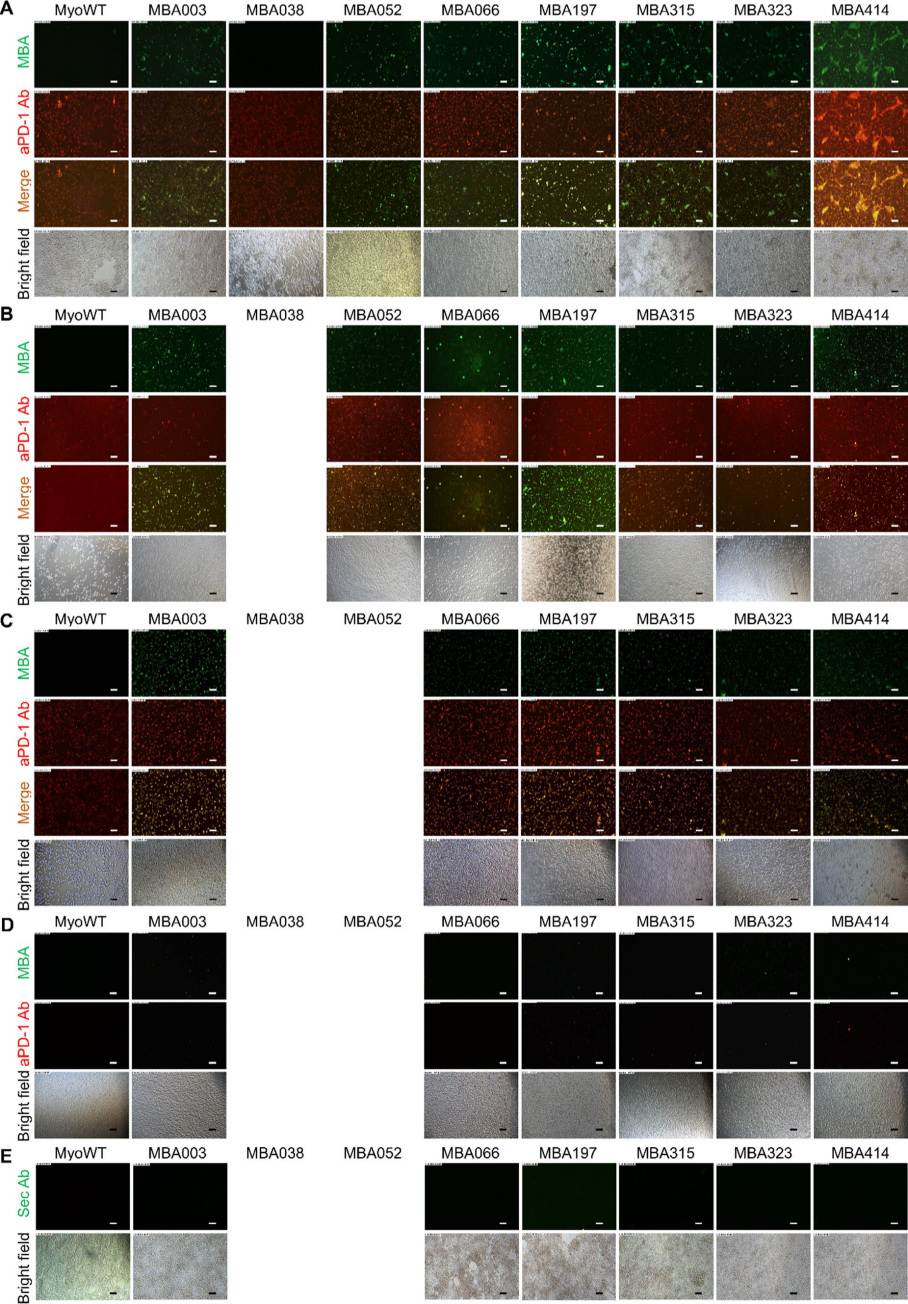
Binding of MBA variants to human cells. **A** HEK293T cells transiently transfected with plasmid carrying cDNA of hPD-1 and stained with selected 8 MBA variants or MyoWT as a control. **B** Binding of 7 selected MBA variants or MyoWT as a control to human acute lymphoblastic lymphoma cells (SUP-T1). **C** Binding of 6 most promising MBA variants and MyoWT to paraformaldehyde-fixed T-cell adult acute lymphocytic leukemia cells MOLT-4. **D** Negative control staining on B cell line Dakiki not expressing PD-1 with selected MBA proteins. **E** hPD-1-transfected HEK293T cells: secondary antibody control staining to results in **A**. The staining with selected MBA proteins was performed without anti-PD-1 primary antibody using the secondary antibody donkey anti-goat Alexa Fluor 488. MBA variants used in figure containing C-terminal V5 tag were detected using mouse monoclonal anti-V5-tag-Alexa Fluor 488 conjugated antibody. Expression of hPD-1 on HEK293T transfected cells as well as on SUP-T1 and MOLT-4 cells was monitored with goat anti-human polyclonal anti-PD-1 antibody followed by secondary donkey anti-goat IgG conjugated with Alexa Fluor 568

HEK293T cells transiently expressing hPD-1 receptor were stained with the selected MBA variants and co-stained with anti-PD-1-specific monoclonal antibody (mAb) to visualize binding of the MBA proteins to surface-expressed hPD-1. Results of immunofluorescence microscopy showed that all the selected MBA variants, except MBA038, exhibited a specific binding to hPD-1-expressing HEK293T cells which correlated with anti-PD-1 mAb staining (Fig. 2A). To further verify the sensitivity and specificity of MBA variants for cell surface hPD-1 staining, we used human acute lymphoblastic lymphoma cells (SUP-T1) which have been reported to express a substantial level of PD-1 on their surface [41]. With the exception for MBA038 variant, double immunofluorescence staining of SUP-T1 cells with all other 7 MBA variants proven their binding to the surface of the SUP-T1 cells in correlation with anti-PD-1 mAb staining (Fig. 2B). Furthermore, hPD-1-HEK293T transfectants, SUP-T1, and MOLT-4 cells showed no staining by fluorescence signal with Myomedin wild-type protein in contrast to anti-PD-1 mAb staining (MyoWT, Fig. 2A–C), revealing the specificity of the tested MBA variants against the hPD-1 receptor. To further confirm the hPD-1 specificity of MBA proteins, we verified binding of 6 MBA variants to human B-cells Dakiki, which lack the expression of hPD-1 and is reported by negative anti-PD-1 polyclonal antibody staining (Fig. 2D). The specificity of secondary antibody conjugate used in Fig. 2A is documented in Fig. 2E.

### Prediction of MBA/PD-1 binding modes by docking

The individual MBA variants were modeled using the amino acid sequence (Additional file 1: Fig. S2a) and docked with hPD-1 model. The protein–protein docking results are summarized in Figure S2b-g showing the first three most probable predicted MBA binding modes for each MBA variant (color coded by decreasing probability from red to yellow). The PD-1 geometry resembles a triangular prism with three relatively flat faces available for interaction. The docking results show a clear preference for interaction with one of the sides and all the most probable predicted modes occupy the same side of the PD-1 model. For variants MBA197, MBA315, and MBA323, the docking procedure also predicts interactions with the second side of PD-1. All the predicted binding modes, however, occupy these two PD-1 sides and only marginally approach the third side responsible for the binding of the PD-L1 described by the available structure and predicted hPD-1/hPD-L1 model (see Additional file 1: Fig. S1).

### Characterization of binding affinity, kinetics, and specificity of MBA variants

**Fig. 3:**
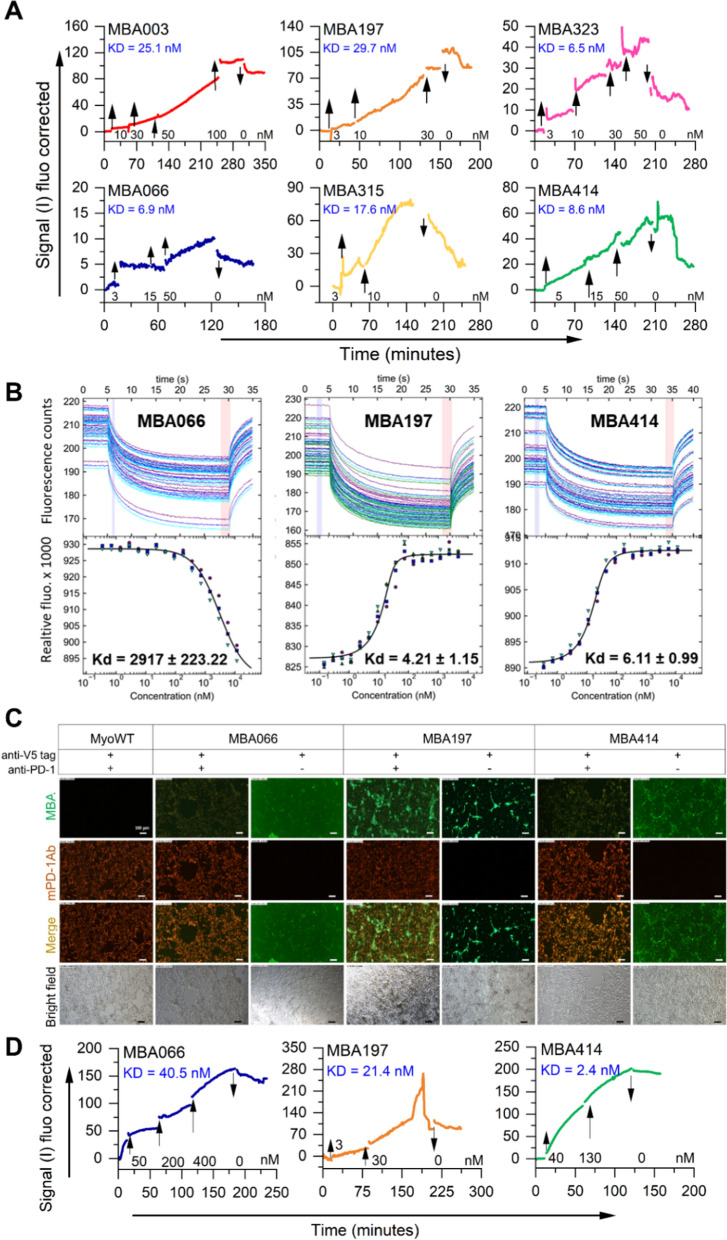
Characterization of binding affinity and specificity of the most important MBA variants. **A** Binding affinity and kinetics measurement for the selected MBA variants to hPD-1 expressed on transfected HEK293T cells using LigandTracer Green Line instrument. **B** Determination of binding dissociation constant of the most promising MBA variants to recombinant hPD-1 by micro-scale thermophoresis. **C** MBA variants tested for binding to murine PD-1 expressed on HEK293T cells. Cells were stained with either both antibodies (anti-V5 tag for MBA recognition and anti-PD-1 mAb for staining of the murine PD-1 receptor) or with anti-V5 tag antibody only. **D** Measurement of binding kinetics and affinity of MBA066, MBA197, and MBA414 to murine PD-1 expressed on HEK293T transfected cells using LigandTracer Green. In **A** and **D**, colored lines indicate signals from cells subtracted from the background

To verify binding affinity and kinetics for 7 MBA variants, HEK293T cells were transfected with pcDNA6 plasmid carrying hPD-1 cDNA. Two days after the transfection, binding kinetics for 7 Myomedin variants was measured using a LigandTracer Green Line instrument. During the measurements, increasing concentrations of the MBA proteins were added to the culture medium, and measurements were collected in real-time until the saturation state was observed. Kinetic on- and off-rates were evaluated for all the measured Myomedin variants. Based on the measurements, the lowest estimated KD was measured for variants MBA323 (6.59 nM), MBA066 (6.92 nM), and MBA414 (8.63 nM). The slowest off-rate was represented by MBA003, MBA066, and MBA197 (kd [1/s] was 1.71 × 10^–5^, 4.63 × 10^–5^, and 7.30 × 10^–5^, respectively (Fig. 3A, Additional file 1: Table S3). In addition to fluorescent microscopy, KD calculation using LigandTracer technique confirmed the binding of all 7 MBA proteins to PD-1–expressing cells. To independently verify the binding affinity, an alternative estimation of Kd values for MBA066, MBA197 and MBA414 variants to recombinant hPD-1 in solution was performed using microscale thermophoresis (MST). Figure 3B indicates considerable Kd values for MBA197 (4.21 ± 1.15 nM) and MBA414 (6.11 ± 0.99 nM), except MBA066 with showed remarkably higher Kd value (2917 ± 223.22).

As we intended to use the most promising MBA variants for in vivo monitoring of PD-1^+^ cell populations in mice, we aimed to verify whether selected MBA variants retain high-affinity binding to murine PD-1 receptor. To this goal, sequence comparison between human and murine extracellular moiety of the PD-1 (Additional file 1: Table S4) indicated a conserved binding consensus important for interaction with PD-L1. After that, HEK293T cells were transiently transfected with pcDNA6 vector carrying murine PD-1 cDNA, and after two days, cells were stained with MBA066, MBA197, and MBA414 proteins. Results presented in Fig. 3C, D and Additional file 1: Table S5 document that all three MBA proteins bind to murine PD-1 transiently expressed on HEK293T transfectants.

### Biodistribution of radiolabeled MBA proteins and in vivo PET/CT imaging

Myomedins variants, named MBA066, MBA197, MBA414, and MyoWT as control, were selected as the most promising candidates for biodistribution analysis in the mouse model. Before radiolabeling of Myomedins, the proteins were conjugated with a chelator Deferoxamine (DFO) using NH_**2**_ group of Myomedin lysine residues, which are naturally presented in this scaffold protein. For conjugation to NH_**2**_ groups, p-SCN-Bz-Deferoxamine was used. For radiolabeling of MBA proteins, ^68^Galium isotope was chosen and ^68^Ga-DFO-MBA variants were dialyzed to PBS. The radiochemical purity of the labeled Myomedins was verified by instant thin-layer chromatography (iTLC) and reached values around 95% (Additional file 1: Table S6), which meets the typical criteria for clinically used diagnostic radiopharmaceuticals. The stability of the radiolabeled Myomedins proteins in human serum was assessed after 30, 60 or 120 min. In vitro, stability of ^68^Ga-MBA066, ^68^Ga-MBA197, and ^68^Ga-MBA414 after 2 h in human serum reaches values between 89.6–98.2% and did not differ from values for ^68^Ga-labeled parental non-mutated Myomedin wild-type protein (95.9–98.0%), as shown in Additional file 1: Table S6.

**Fig. 4:**
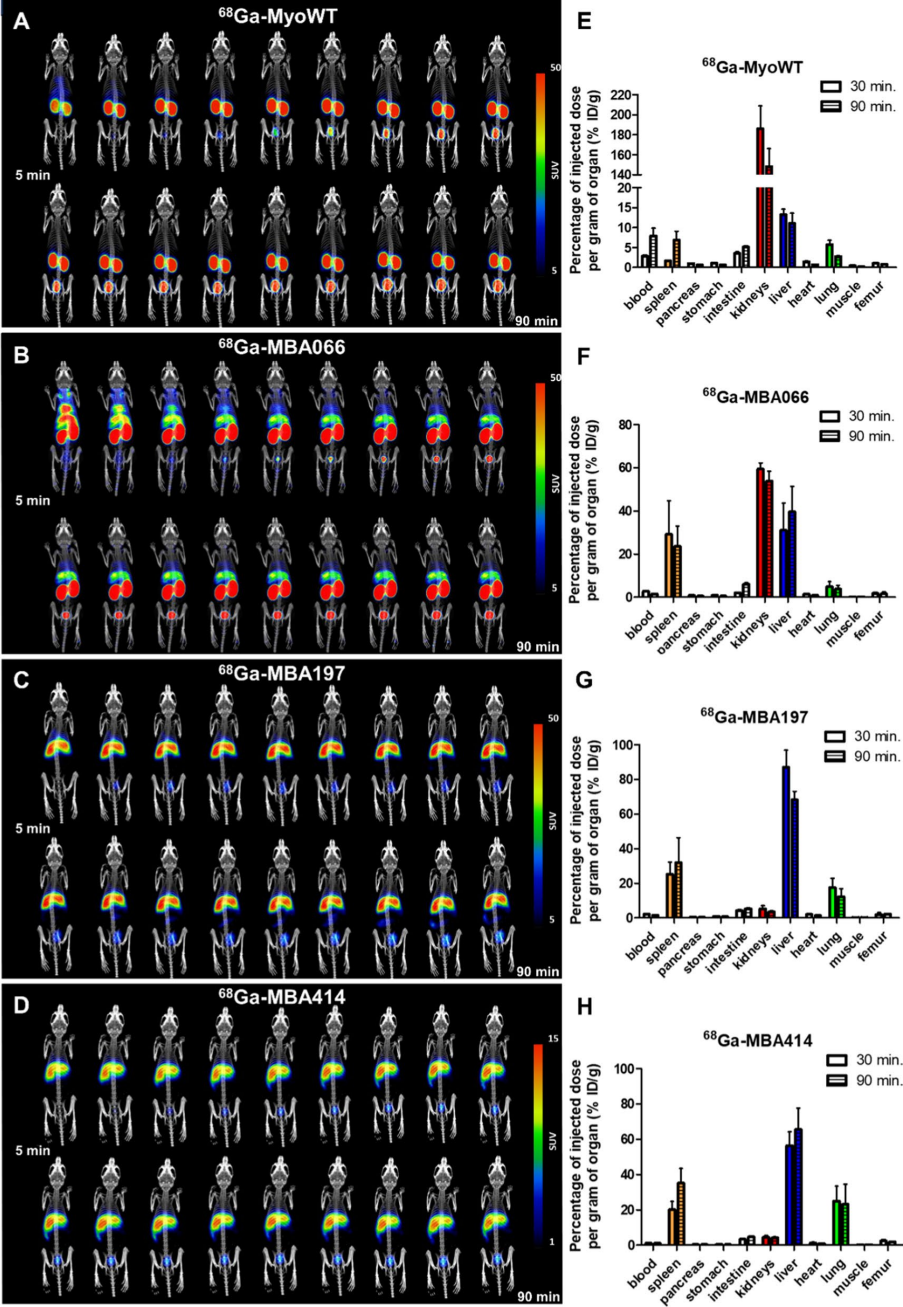
In vivo imaging and ex vivo analysis of ^68^Ga-labeled MBA distribution in Balb/c mice. **A** ^68^Ga-MyoWT, **B** ^68^Ga-MBA066, **C** ^68^Ga-MBA197, **D** ^68^Ga-MBA414. Mice were retro-orbitally injected with particular MBA binders and immediately scanned by a PET/CT scanner. PET imaging was performed in the form of eighteen consecutive 5-min PET scans. **E** ^68^Ga-myoWT,** F** ^68^Ga-MBA066, **G** ^68^Ga-MBA197, **H** ^68^Ga-MBA414. Mice were retro-orbitally injected with ^68^Ga-binders (*n* = *3* mice per group). Blood, spleen, pancreas, stomach, intestines, kidneys, liver, heart, lungs, muscle, and bone were collected at 30 and 90 min after injection. The tissue samples obtained were weighed, and radioactivity was measured in a γ-counter. The biodistribution data were calculated as the percentage of injected dose per gram of tissue (% ID/g)

In vivo, the biodistribution of radiolabeled MBA066, MBA197 and MBA414 proteins in comparison to MyoWT parental non-mutated protein was tested in BALB/c mice. After retro-orbital injection of ^68^Ga-Myomedins, the distribution of proteins was monitored using whole-body positron emission tomography combined with computerized tomography (PET/CT) imaging up to 90 min post-injection. Results presented in Fig. 4A demonstrate that ^68^Ga-MyoWT is quickly accumulated in the kidneys and excreted from the body via the bladder. This indicates that parental MyoWT did not substantially accumulate in any particular organs, except excretory system, and therefore, can be used as a negative control for in vivo PET/CT imaging. In the case of MBA066, a rapid accumulation of radiolabeled protein is visible in the heart, where it is presented during first 25 min monitoring period. The substantial amount of signal is detected in the liver for all monitoring duration (Fig. 4B). In striking contrast to MyoWT, both MBA197 and MBA414 exhibited a substantial accumulation in the liver and remained concentrated there for entire monitoring period, with only a slow clearance via kidneys into the bladder (Fig. 4C, D). This suggests a specific interaction to PD-1^+^ cell population mostly in the liver, but also in a lower content in other tissues.

Furthermore, to investigate more precisely the biodistribution of PD-1 binding Myomedins, we performed *post mortem *ex vivo analysis of ^68^Ga-Myomedins content in multiple mouse organs. In this study, quantification of radiolabeled MBA066, MBA197, MBA414, and MyoWT proteins after 30 or 90 min of administration was assessed in peripheral blood and other 10 organs of interest in mice. As shown in Fig. 4E, MyoWT is substantially accumulated in the kidneys and weakly observed in liver, spleen, and blood. In contrast to MyoWT organ distribution, MBA197 and MBA414 were prominently concentrated in liver (6–7 times), spleen (4–7 times), and lung (8–10 times) (Fig. 4G, H). In addition, MBA197 and MBA414 did not exhibit significant accumulation in other organs, such as kidneys, pancreas, stomach, heart, muscle, femur, and blood (Fig. 4G, H). These observations suggested that MBA197 and MBA414 could specifically interact with PD-1-expressing cells and, due to high affinity binding, thus remain concentrated in organs with substantial lymphocyte populations, at least for the monitored 90 min period.

To further support this hypothesis, we used a mouse muscle infection model in which lymphocyte homing is stimulated by a local intramuscular application of bacterial suspension into the mouse leg. Therefore, *E. coli* suspension (5 × 10^7^ CFU) was administrated 5 h before the PET/CT imaging of Myomedin proteins was initiated. As presented by a non-infected dynamic biodistribution study on the ^68^Ga-MBA066 variant (Fig. 4B), this protein also predominantly binds to liver cell populations, but in comparison to MBA197 and MBA414 variants, it is faster cleared via kidneys, probably due to lower binding affinity. When ^68^Ga-MBA066 protein was administrated in the mouse infection model, a distinct local signal concentration, in comparison to ^68^Ga-MyoWT control, was detected in the site of infection 45 min post-injection (Additional file 1: Fig. S3). Thus, ^68^Ga-MBA066 exhibited a slower pharmacokinetics and increased local concentration in contrast to ^68^Ga-MyoWT. This further supports the expectation that ^68^Ga-MBA066 might specifically recognize PD-1^+^ lymphocytes expectedly homing into the site of infection.

### Staining of PD-1 expressing cells with MBA proteins on frozen sections of human tonsils and NSCLC tissues

**Fig. 5:**
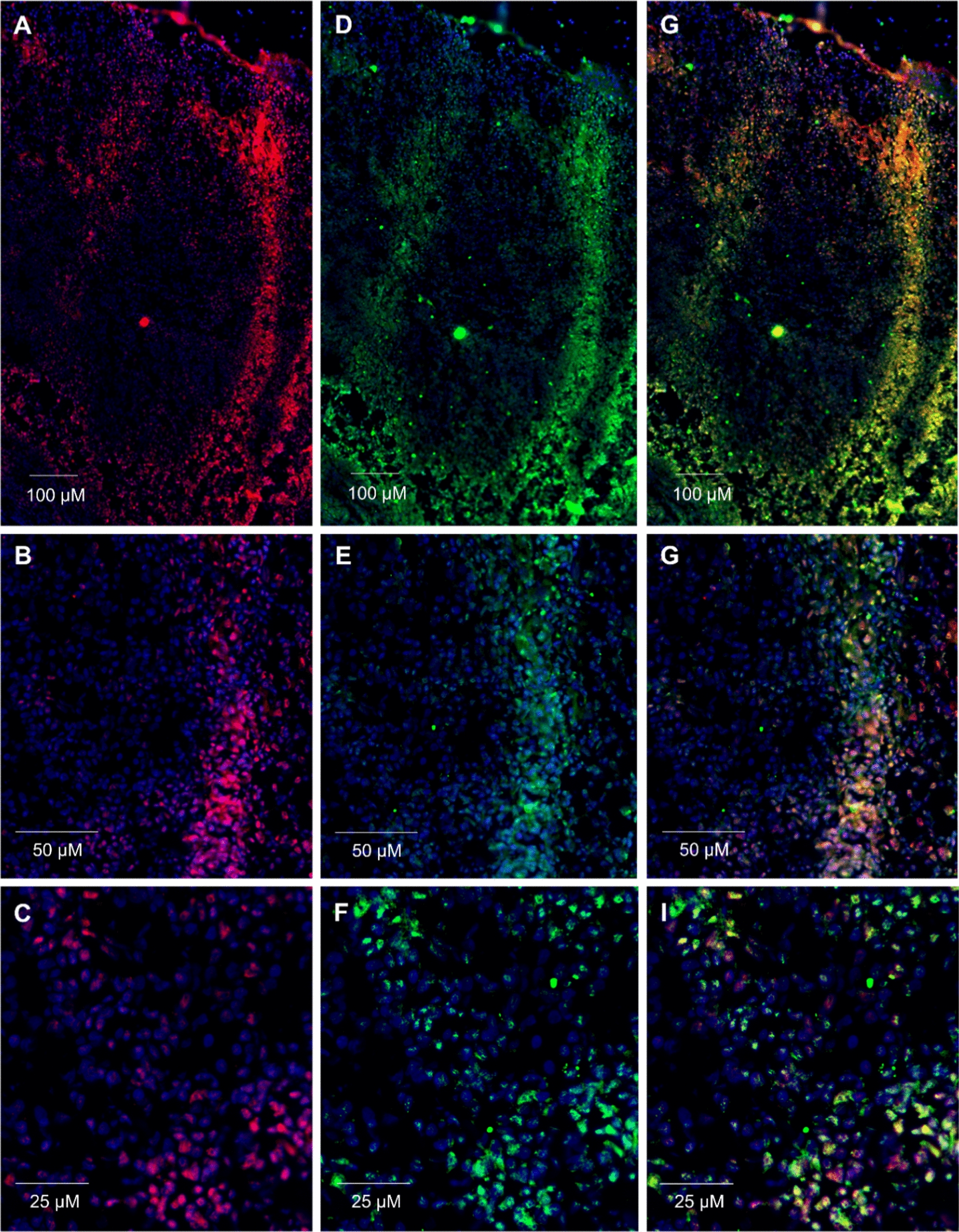
Immunofluorescence staining of PD-1^+^ T cells on human tonsil frozen sections. **A**–**C** Tissue was stained with MBA414 variant detected by anti-V5 IgG-Alexa Fluor 647 antibody. **D**–**F** Staining with rabbit anti-PD-1 IgG-FITC antibody. **G**–**I** Merge of **A** and **D**, **B** and **E**, **C** and **F** demonstrate superimposed fluorescence signals. Magnifications of tissues sections are ×200 **A**, **D**, **G**, ×400 **B**, **E**, **H** and ×1000 **C**, **F**, **I**. Significant co-localization of MBA414 variant detected by anti-V5 IgG-Alexa Fluor 647 antibody (**A**) and rabbit anti-PD-1 IgG-FITC (**D**) was analyzed through Pearson´s correlation coefficient (r) calculated by ImageJ software with JACoP co-localization tool, r = 0.779

**Fig. 6:**
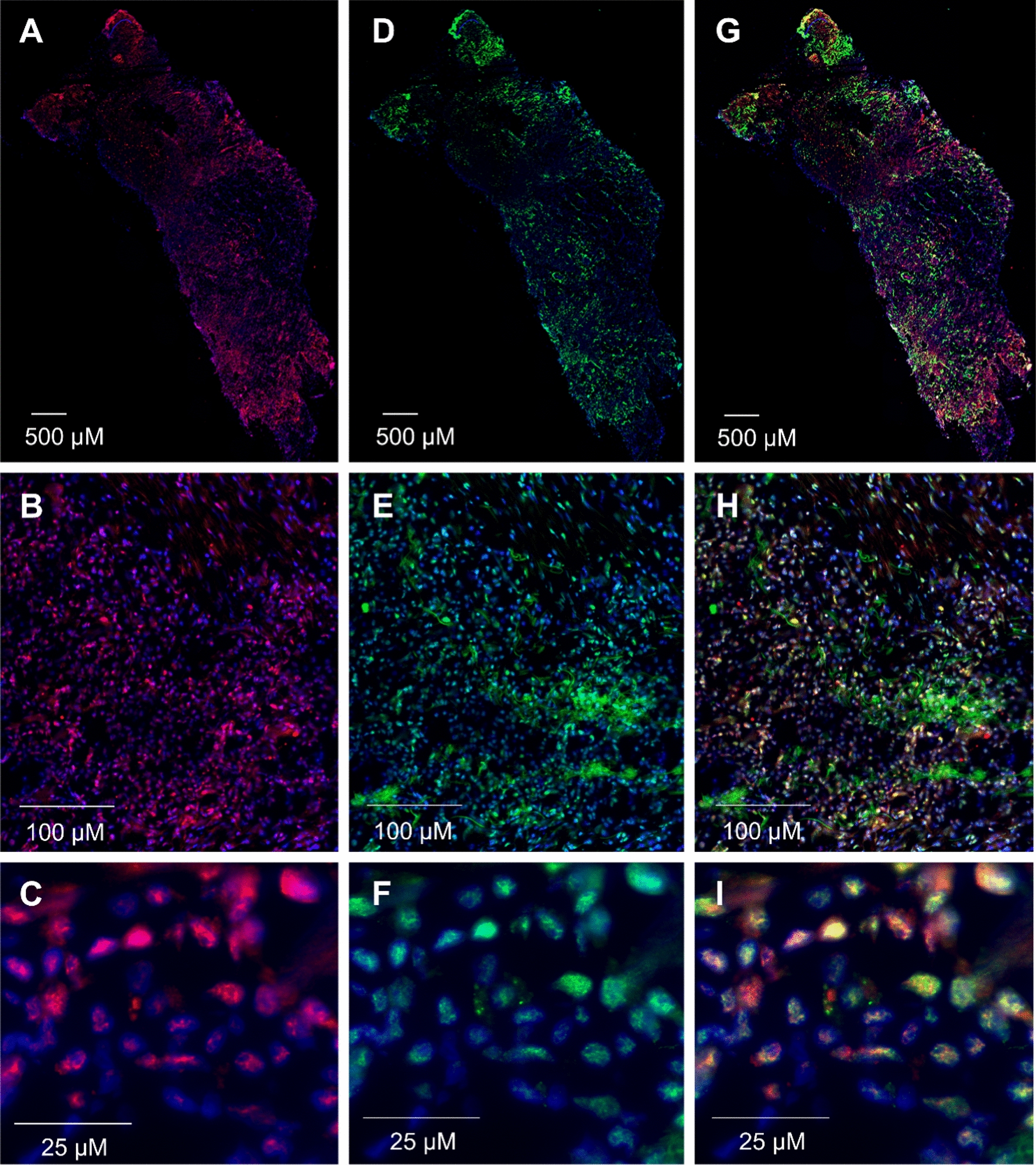
Immunostaining of PD-1^+^ cells on NSCLC tissue. **A**–**C** Frozen sections of tumor tissue were stained with MBA414 variant detected by anti-V5 IgG-Alexa Fluor 647 conjugate and **D**–**F** rabbit anti-PD-1 IgG-FITC conjugated antibody. Panel **G**–**I** merge fluorescence signals of MBA414 (Alexa Fluor 647) and anti-PD-1 IgG (FITC). Magnifications of tissue sections are ×200 (**A**, **D**, **G)**, ×400 (**B**, **E**, **H**) and ×1000 (**C**, **F**, **I)**. Significant co-localization of MBA414 variant detected by anti-V5 IgG-Alexa Fluor  647 antibody (**A**) and rabbit anti-PD-1 IgG-FITC (**D**) was analyzed through Pearson´s correlation coefficient (r) calculated by ImageJ software with JACoP co-localization tool, r = 0.846

To prove the binding ability of MBA414 to PD-1 in normal human tissue, we used a human tonsil resected from a child with tonsillar hypertrophy. In tonsils, B cells follicles are surrounded by T cell zones. The PD-1 receptor is expressed here preferentially on activated T cells,  B cells, dendritic cells, macrophages, monocytes, and NK cells [42, 43]. IHC staining proved the ability of MBA414 to recognize PD-1^+^ cells with pattern of staining similar to that pattern obtained with rabbit polyclonal anti-PD-1 antibody (Fig. 5). Double-positive cells are clearly identifiable after merging fluorescence signals from MBA414-Alexa Fluor 647 and anti-PD-1 IgG-FITC (Fig. 5G–I). Next, we proofed MBA414 binding on a frozen NSCLC tissue section (Fig. 6). In analogy to tonsillar tissue (Fig. 5), PD-1 expression on NSCLC sections was detected using MBA414 variant and rabbit polyclonal anti-PD-1 antibody. Merged pictures show an overlap of fluorescence signals from MBA414-Alexa Fluor 647 and anti-PD-1 IgG-FITC (Fig. 6G–I). The comparison indicates that MBA414 detects more cells than the anti-PD-1 antibody, which corresponds to our expectations.

## Discussion

Tumor-infiltrating lymphocytes (TILs), also known as the “immunoscore” [44, 45], are characterized as lymphocytes within and around cancer cells in tumor microenvironment (TME) and have been associated with better prognosis, and correlated with the traditional tumor-node-metastasis (TNM) staging and microsatellite instability (MSI) status in cancer patients [46]. Moreover, TILs were also used to envisage the clinical benefit of anti-PD1 immunotherapy, especially at the invasive margin where they were directly correlated with response to anti-PD-1 immunotherapy and an immunohistochemistry-based assessment of CD8^+^ T cell density in the core of the tumor [47, 48].

Among the dominant challenges for the laboratory diagnosis of PD-1 and its cognate PD-L1 counterpart in tumor tissue samples are the highly variable expression dynamics within different areas of the same tissue, the lack of reliable antibodies and diagnostic kits for some types of solid tumors, the expression heterogeneity of both biomarkers in the context of TILs and tumor cells, and above all, insufficient evidence of a correlation between PD-1 or PD-L1 expression and the treatment response. This makes the assessment of cutoff points rather difficult. A typical case, for example, is colorectal carcinoma, for which, until now, there has been no definite scoring system developed. The above-mentioned factors can be the cause of why patients with low PD-1 and PD-L1 expression as assessed by current histopathological scoring methods can benefit from immunotherapy. This could be a reason why some centers give immunotherapy to all patients based on their current clinical performance status.

Employment of identified MBA binders could offer an alternative to antibody-based diagnostics applicable for in vitro and in vivo testing (Additional file 1: Table S7). In the case of NSCLC in vitro diagnostics, MBA414 variant exhibited similar PD-1 positivity pattern as available anti-PD-1 mAb in fluorescence microscopy (Fig. 6; Pearson’s correlation coefficient 0.846). This is promising for testing of MBA binders in large cohort NSCLC biopsies and also other tumors. Nevertheless, more accurate assessment of the in vitro diagnostic potential of MBA requires further testing, particularly in studies evaluating the potential to predict response to anti-PD-1 immunotherapy. Due to a small size of MBA, we could expect an improved penetration into the tumor tissue in comparison to mAb. Thus, it is also possible to hypothesize that radiolabeled MBA could offer an enhanced sensitivity of tumor PD-1 expression analyses for PET/CT body imaging techniques.

Another point to consider is that in routine histopathological practice, at least in most countries, the expression of the above-mentioned factors is examined from primary tumors, and physicians treat the metastatic disease. It is generally known that the expression of these factors differs in primary tumor and metastasis. Also, the area of the examined tissue does not represent the whole tumor. The most important point to consider is that the histopathologic assessment represents a static picture and does not reflect the dynamics of tumor development and consequent reaction of the immune system.

Although unprecedented success has been achieved in the diagnosis and treatment of cancer patients with antibodies, non-antibody biologics have attracted considerable attention [49, 50]. Based on our knowledge, only one small-size protein scaffold library (repebody) [51], one de novo-designed hyperstable miniprotein PD1 agonist [52], and two affinity-optimized soluble human PD-L1 (L3B3-hPD-L1 and L3C7-hPD-L1) [53], have been proven as efficient non-antibody anti-PD-1 biologics. Additionally, several bioactive small-molecules and peptide-based inhibitors as potential alternatives to mAbs targeting PD-1 protein are also reported (reviewed in Refs. [54, 55]). In considering small proteins for mapping PD1^+^ TILs using immuno-PET imaging, their small size provides an enhanced tumor penetration, together with an increased glomerular filtration and renal losses [14]. Furthermore, PET imaging can be used for a repeated assessment of the tumor growth progression, thereby completing diagnostic information reached by immunohistochemical approaches [14].

The primary goal of our study was to identify high-affinity and high-specificity PD-1 binding proteins for ex vivo diagnostics and in vivo imaging; however, the used library concept with the randomization of 12 amino acid residues on β-sheet surface enabled us to also identify potential PD-1 blocking variants. Indeed, two of the most promising variants, MBA003 and MBA323, competed with a soluble form of the human PD-L1 for binding to hPD-1 (Additional file 1: Figs. S4, S5, S6). Based on competition ELISA data, we can estimate the binding affinity for the PD-L1 interaction binding site. For the MBA003 variant, the estimated binding affinity is 10 µM and for MBA323 is 30 µM. These values are comparable to the modest 8.2 µM binding affinity described for the extracellular moiety of natural human PD-L1 [56]. To independently confirm the inhibitory potential of MBA003 and MBA323 proteins, we treated hPD-1-transfected HEK293T cells with 50 nM human PD-L1, then incubated with 40 µg/ml MBA variants (and MyoWT control) and fluorescently detected the binding in the presence or absence of the human PD-L1 competitor. Also, we performed the staining with anti-PD-1 antibody to verify a co-localization of the signal. As expected in the correlation to competition ELISA data (Additional file 1: Fig. S4), the binding of MBA003 and MBA323 to hPD-1-HEK293T transfectants was substantially diminished in comparison to non-competing variants MBA066, MBA197, and MBA414 (Additional file 1: Fig. S5).

Furthermore, we performed a competition binding assay using LigandTracer. We compared binding curves for competing variants MBA003 and MBA323 in the presence of hPD-L1 with those for non-competing variants MBA066 and MBA414 using hPD-1-transfected HEK293T cells (Additional file 1: Fig. S6). Results again confirm an overlapping binding site of MBA003 and MBA323 with competing hPD-L1 on the hPD-1 receptor. Thus, we complete data of three different molecular approaches—competition ELISA, competition fluorescent microscopy, and competition real-time imaging by LigandTracer method. These two MBA proteins (MBA003, MBA323), therefore, could be further improved to increase a blocking potential by affinity maturation approaches.

Based on structural data (PDB ID 3bik), the overall shape of the PD-L1 interacting face is partially concave due to the surrounding hairpin/loop regions. It was shown that these outer regions contribute to receptor/ligand recognition and can be considered as an allosteric region of the PD-1 protein [57]. While our model of the hPD-1/hPD-L1 shows clear differences in geometry of the previously described CC’ and FG loops, the largest rearrangement was observed for the loop formed by residues E84 to R94. As our selection approach was not set up for a screening of PD-L1 competing variants, we rather identified high-affinity non-competing variants in the KD range 6.59–29.7 nM (Additional file 1: Table S3). This is consistent with the most probable binding modes of all six MBA variants predicted by docking, occupying the PD-1 face not involved in the PD-L1 interaction (Additional file 1: Fig. S2, Table S4).

The crystal structures of murine PD-1 and human PD-L1 complexes (PDB ID 3bik and 3sbw) revealed a contact interface involving 16 residues of mPD-1 and 14 residues of hPD-L1 [58]. There is 64% protein sequence identity between murine and human PD-1 (Additional file 1: Table S4), which can cross interact with PD-L1 of both species with similar affinities [59–61]. Indeed, the estimated KD value for MBA414 variant measured by cell surface interaction with hPD-1 on HEK293T transfectant cells (KD = 8.6 nM, Fig. 3A) correlates with that for murine PD-1 (KD = 2.48 nM, Fig. 3D, Additional file 1: Table S5). MBA197 binds to hPD-1 with similar binding affinity as compared to murine PD-1 (KD = 29.7 nM versus KD = 21.4 nM, respectively, Fig. 3A, D, Additional file 1: Table S5). However, in the case of MBA066, the affinity to hPD-1 (KD = 6.9 nM, Fig. 3A) is substantially decreased for murine PD-1 (KD = 40.5 nM, Fig. 3D, Additional file 1: Table S5). Such a loss of binding affinity for murine PD-1 could explain the observed difference in biodistribution time course in mice (Fig. 4B) with an increased kidney clearance in contrast to MBA197 and MBA414 with a non-exacerbated affinity for murine PD-1.

To assess the penetration of MBA proteins into tissues overexpressing PD-1, we employed a murine model of local acute immune system activation based on intramuscular injection of viable *E. coli* cells. During immune activation, PD-1 is overexpressed on many cell types including macrophages, dendritic cells, CD4^+^ and CD8^+^ T cells, NKT cells, and B cells. During bacterial infection, PD-1 expression could be induced by antigen-dependent stimulation such as IgM crosslinking on B cells, TCR-mediated activation of CD4^+^ T cells, by bacteria pathogen-associated molecular patterns, such as lipopolysaccharide, which in mice activates both B cells and macrophages, or by inflammatory cytokines IL-1β, IL-6, and TNF-α [62, 63]. PD-1 expression could be induced by NFATc1 and/or NF-κB pathways of activation. After the comparison with wild-type non-randomized Myomedin parental protein (MyoWT), a significant increase in the accumulation of ^68^Ga-MBA066 was detected at the site of injection (Additional file 1: Fig. S3).

Small protein-derived diagnostics and therapeutics are of a hot focus. Those include nanobodies-based PD-1-Nb-B20 as small IgG-derived protein targeting linear PD-1125–136 (AISLAPKAQIKE) peptide [64]. This small protein was developed for the therapeutic purpose with only a limited characterization (the specificity by flow cytometry, binding to PD-1 reduced by 84.8, 93.5, and 93.9% in BxPC-3 cells after treatment with 1, 10, and 100 µg/ml of nanobody, respectively) [64]. Also, Miyazaki et al. demonstrated peptide-barcoded nanobody (PBNb) targeting PD-1 with PBC1, PBC2, and PBC9 candidates with the 1–52 nM affinity estimated by ELISA [65]. Zhang et al. [66] developed anti-PD-1 Nb-Fc nanobody targeting PD-1 with 6.6 nM affinity measured by SPR. Ding et al. [67] reported nanobody-based trispecific T cell engager (Nb-TriTE) generated with KD values measured by SPR for hPD-1 as 782 nM, for human CD3 as 104 nM, and human fibroblast activation protein as 77.7 nM. In summary, all PD-1–specific nanobodies were developed primarily for therapeutic, not diagnostic purpose. Some candidates were characterized only partially using limited biophysical approaches and were not tested for in vivo imaging nor ex vivo diagnostic reliability. In contrast to nanobodies with the randomization of protein loop residues, our non-immunoglobulin cysteine-free Myomedin scaffold concept provides different geometry by randomization of β-sheet residues that resulted in a portfolio of 6 MBA variants with high-affinity to hPD-1 for diagnosis or blocking effect. Myomedins with 13 kDa molecular weight provide an excellent tissue penetration, sufficient sensitivity for frozen tissue sections staining and cheap prokaryotic production costs compared to the immunoglobulin molecules.

In this manuscript, we analyzed the distribution of ^68^Ga-labelled MBAs for up to 90 min via ex vivo biodistribution study and dynamic PET/CT imaging. The injected substances were well tolerated for several days after application as the mice used for the imaging study were sacrificed approximately 2 weeks after the imaging without clinical and neurological marks of alterations. In support, we have previously published experiments on Myomedin-derived binders (each containing 10 µg) for immunization (in Freund adjuvant) through intradermal application of experimental BALB/c mice [34, 39]. We did not record any marks of somatic or behavioral alterations, with exception of local irritation due to adjuvant co-administered with individual Myomedins. Also, we tested whether the sera from Myomedin-immunized mice recognize murine cellular antigens to investigate the potential of Myomedins acting as an autoantigens (Additional file 1: Fig. S7). We incubated pooled Myomedin-immunized sera (dilution 1:50) and naive sera with Triton-X100-permeabilized murine fibroblasts NIH 3T3 followed by IgG binding detection with Alexa Fluor 488-labeled anti-IgG antibody. In Additional file 1: Fig. S7, no detectable reaction of Myomedin-immunized mice sera with cell antigens was noticed. As a positive control, we used formerly collected sera from mice immunized with murine heat shock protein 70 kDa (Hsp70). In contrast to no reactivity of Myomedin-immunized mice, the sera from Hsp70-immunized mice recognized moderately the intracellular antigens, according to observed pattern probably inside mitochondrion or endoplasmic reticulum (Additional file 1: Fig. S7).

Collectively, we present here a concept of highly complex β-sheet combinatorial library developed on Myomedin scaffold as a valuable alternative for the generation of small protein binders for biomedical applications. Randomization of amino acid residues on a flat β-sheet surface provides a proof-of-concept for the selection of high-affinity binders and, thus, completes a portfolio of loop-type scaffold libraries [34, 39, 51] and three-helix bundle-based libraries [68–70]. Herein, we provide evidence that β-sheet-derived MBA variants exhibit PD-1 specificity and stability in human serum. The binding affinity of the best MBA-series candidates is sufficient for selective in vitro diagnostics of tumor tissue biopsies and sensitive for in vivo PET/CT imaging. Due to small size of Myomedin binders, which allows an increased tumor tissue penetration, the engineered MBA protein variants should be beneficial for monitoring PD-1^+^ cell populations in solid tumors, including NSCLC.

### Supplementary Information

**Additional file1.** **SI Methodology:** Screening of PD-1 specific variants by ELISA; Production and purification of MBA variants; Cell cultures; DNA plasmid preparation and transfections of HEK293T cells; Transfection of HEK293T cells for immunofluorescence staining; Transfection of HEK293T cells for binding affinity measurement using LigandTracer; Detection of MBA binding to cell surface by immunofluorescence staining; Binding kinetics of MBA proteins measured with Ligand Tracer and competition assay; Competition ELISA; Determination of K_d_ by micro-scale thermophoresis (MST); **SI Results:** Figure S1. Summary of experimental and predicted geometries of PD-1/PD-L1 complexes. Table S1. List of primers used for assembly of myomedin beta sheet combinatorial library where XXX indicates randomized position. Figure S2. List of sequences of selected MBA variants and summary of most probable predicted PD-1 binding modes. Table S2. Criteria for selection of MBA variants for experiments performed on (A) tissue sections and in vivo experiments performed on (B) mice. Table S3. Estimation of kinetic parameters for six MBA variants binding to human PD-1 cDNA-transfected HEK293T cells analyzed by LigandTracer Green. Table S4. Sequence comparison between extracellular part of human and mouse PD-1. Table S5. Estimation of kinetic parameters for three MBA variants binding to murine PD-1 cDNAtransfected HEK293T cells analyzed by LigandTracer Green. Table S6. Radiochemical purity of 68Ga-binders and in vitro stability in human serum. Figure S3. Distribution of 68Galium-labeled MBA066 and MyoWT proteins in *E. coli* infected Balb/c mice. Figure S4. Competition of MBA proteins with human PD-L1 for binding to PD-1 using ELISA. Figure S5. Competition of MBA proteins with human PD-L1 for binding to PD-1-transfected HEK293T cells. Figure S6. Competition of MBA proteins with hPD-L1 for binding to hPD-1 using LigandTracer. Table S7. Comprehensive overview of MBA Myomedin variants used in this study. Figure S7. Myomedins do not elicit autoantibodies after immunization of experimental mice.

## Data Availability

All data generated or analyzed during this study are included in this article and its Supplementary Information/Source Data file. Source data are (will be) provided with this paper. The coordinates from ClusPro protein–protein docking and PyMOL session summarizing the results are available from the zenodo repository ( 10.5281/zenodo.8182102).
