# Supplementary material for: Engineering PD-1-targeted small protein variants for in vitro diagnostics and in vivo PET imaging
Source: J Transl Med. 2024 May 6;22:426. doi: 10.1186/s12967-024-05210-x (PMC11071268; doi:10.1186/s12967-024-05210-x)
Supplement: Supplementary file 1 — Additional file1. SI Methodology: Screening of PD-1 specific variants by ELISA; Production and purification of MBA variants; Cell cultures; DNA plasmid preparation and transfections of HEK293T cells; Transfection of HEK293T cells for immunofluorescence staining; Transfection of HEK293T cells for binding affinity measurement using LigandTracer; Detection of MBA binding to cell surface by immunofluorescence staining; Binding kinetics of MBA proteins measured with Ligand Tracer and competition assay; Competition ELISA; Determination of Kd by micro-scale thermophoresis (MST); SI Results: Figure S1. Summary of experimental and predicted geometries of PD-1/PD-L1 complexes. Table S1. List of primers used for assembly of myomedin beta sheet combinatorial library where XXX indicates randomized position. Figure S2. List of sequences of selected MBA variants and summary of most probable predicted PD-1 binding modes. Table S2. Criteria for selection of MBA variants for experiments performed on (A) tissue sections and in vivo experiments performed on (B) mice. Table S3. Estimation of kinetic parameters for six MBA variants binding to human PD-1 cDNA-transfected HEK293T cells analyzed by LigandTracer Green. Table S4. Sequence comparison between extracellular part of human and mouse PD-1. Table S5. Estimation of kinetic parameters for three MBA variants binding to murine PD-1 cDNAtransfected HEK293T cells analyzed by LigandTracer Green. Table S6. Radiochemical purity of 68Ga-binders and in vitro stability in human serum. Figure S3. Distribution of 68Galium-labeled MBA066 and MyoWT proteins in E. coli infected Balb/c mice. Figure S4. Competition of MBA proteins with human PD-L1 for binding to PD-1 using ELISA. Figure S5. Competition of MBA proteins with human PD-L1 for binding to PD-1-transfected HEK293T cells. Figure S6. Competition of MBA proteins with hPD-L1 for binding to hPD-1 using LigandTracer. Table S7. Comprehensive overview of MBA Myomedin variants used in this study. Fig [file 12967_2024_5210_MOESM1_ESM.docx]

**Supplementary information**

**Engineering PD-1-targeted small protein variants for *in vitro* diagnostics and *in vivo* PET-imaging**

Joanna Maria Mierzwicka^1^*, Hana Petroková^1^*, Leona Rašková Kafková^2,3^, Petr Kosztyu^2,3^, Jiří Černý^4^, Milan Kuchař^1^, Miloš Petřík^5^, Kateřina Bendová^5^, Kristýna Krasulová^5^, Yaroslava Groza^1^, Lucie Vaňková^1^, Shiv Bharadwaj^1^, Natalya Panova^1^, Michal Křupka^2,3^, Jozef Škarda^2,3,6^, Milan Raška^2,3#^, Petr Malý^1#^

^1^Laboratory of Ligand Engineering, Institute of Biotechnology of the Czech Academy of Sciences, BIOCEV Research Center, Průmyslová 595, 252 50 Vestec, Czech Republic

^2^Department of Immunology, University Hospital Olomouc, Zdravotníků 248/7, 77900 Olomouc, Czech Republic

^3^Department of Immunology, Faculty of Medicine and Dentistry, Palacky University Olomouc, Hněvotínská 3, 779 00, Olomouc, Czech Republic

^4^Laboratory of Structural Bioinformatics of Proteins, Institute of Biotechnology of the Czech Academy of Sciences, BIOCEV Research Center, Průmyslová 595, 252 50 Vestec, Czech Republic

^5^Institute of Molecular and Translational Medicine, Faculty of Medicine and Dentistry and Czech Advanced Technology and Research Institute, Palacky University Olomouc, Hněvotínská 5, 779 00 Olomouc, Czech Republic

^6^Institute of Clinical and Molecular Pathology, Faculty of Medicine and Dentistry, Palacky University Olomouc, Hnevotinska 3, 779 00, Olomouc, Czech Republic

# **SI Methodology**

## ***Screening of PD-1 specific variants by ELISA***

MBA variants were produced in *E. coli* BL21 (*DE3*) strain incubated in LB medium with kanamycin (60 μg/ml at 37 °C. Protein expression was induced by adding 1 mM IPTG when OD_600_ reached 0.6 and further incubation was done at 37 °C for an additional 4 h. Centrifuged pellet was either lysate using B-PER® Bacterial Protein Extraction Reagent (Thermo Scientific) or sonicated and purified.

All ELISA assays were performed on MAXISORP Nunc 96-well plates. For each assay, the plate was coated with 1 μg/ml of PD-1 protein (R&D Systems) and incubated in a coating buffer for one hour at room temperature. Then plate was washed with PBST (PBS pH 7.4 with 0.05% Tween-20) and blocked overnight with 1% BSA in PBST at 4 °C. Bacterial cell lysates prepared using B-PER® Bacterial Protein Extraction Reagent (Thermo Scientific) were 50 times diluted in PBST and applied on the blocked plate. Proteins from the Myomedin beta-sheet library containing V5-tag were detected with anti-V5 HRP-conjugated antibody in dilution 1:10 000 (antibodies – online).

## ***Production and purification of MBA variants***

MBA proteins were produced in *E. coli* BL21 (*DE3*) strain incubated in LB medium with kanamycin (60 μg/ml at 37 °C. Protein expression was induced by adding 1 mM IPTG when OD_600_ reached 0.6 and further incubation was done at 37 °C for an additional 4 h. Bacterial cell pellet of 20-200 ml LB culture was sonicated in 50 mM Tris, 150 mM NaCl buffer pH 8.0 and centrifuged for 20 min in 40,000 ×g at 4 °C. Cytosolic extract was purified using Ni-NTA-agarose column under native conditions 50 mM Tris, 300 mM NaCl pH 8.0 and 20 mM Imidazole pH 8.0 (TNI20). When protein was produced in the inclusion bodies, 8 M urea in TNI20 buffer was used for extraction from the bacterial cells which was followed by capture of the MBA protein on Ni-NTA-agarose column under denaturing conditions TNI20 and 8 M Urea, refolding on column by extensive washing using TNI20 buffer and elution under native condition with 50 mM Tris, 300 mM NaCl pH 8.0 and 250 mM Imidazole pH 8.0. The presence of the protein in individual fractions was confirmed by SDS-PAGE. For SDS-PAGE, samples were mixed with 5x Laemmli buffer and heat denatured at 95 °C for 10 min. Following, protein samples were loaded on the polyacrylamide gel along with a molecular weight marker (Dual color, Bio-Rad). Electrophoresis was running for 60 min at constant 35 mA (for one gel), then gel was stained with Coomassie Blue.

## ***Cell cultures***

Human embryonic kidney cells (HEK293T) were cultured in DMEM medium (Biosera) supplemented with 10% fetal bovine serum (FBS) and streptomycin-penicillin solution (Biosera). Acute lymphoblastic lymphoma (SUP-T1) cells (ATCC; CRL-1942) were cultured in RPMI medium supplemented with 10% FBS and streptomycin-penicillin solution. Cells were tested for Mycoplasma presence (Invivogen).

## ***DNA plasmid preparation and transfections of HEK293T cells***

Full human and murine PD-1 (hPD-1 and mPD-1) sequences were derived from UniProt (entry: Q15116 for hPD-1 and Q02242 for mPD-1), synthetized at ThermoFisher Scientific and inserted into pcDNA6 myc-His (version A) plasmid (Invitrogen) using HindIII and XbaI restriction sites which were added to the PD-1 sequence.

## ***Transfection of HEK293T cells for immunofluorescence staining***

Transient transfection of HEK293T cells was done with the use of cationic polymer Polyethylenimine (PEI branched, MW 25,000; Sigma-Aldrich) in concentration 1 mg/ml, 3:1 PEI to plasmid DNA ratio and 1 μg of DNA per 1.5 x 10^6^ cells (when cells were seeded 48h before transfection) or per 0.25 x 10^6^ cells (when cells were seeded 24 h before transfection) on 24 well plates (TPP Techno Plastic Products AG). Right before transfection medium was exchanged into DMEM without any supplements.

Transfection mixes containing PEI and DNA were prepared in 50 μl of DMEM medium without supplements, mixed by inversion, incubated for 15 min at room temperature and then applied dropwise into the wells. Transfected cells were incubated in 0.5 ml of DMEM medium without supplements for 4 h and then 0.5 ml of complete culture medium was added to each well.

## ***Transfection of HEK293T cells for binding affinity measurement using LigandTracer***

HEK293T cells were transiently transfected with 1mg/ml PEI (MW 25,000) and PEI–DNA ratio was 3:1. 24 h before the transfection 3 ml of cell suspensions (1 × 10^6^ cells) were seeded on 87 mm cell dishes in the designated area (marked as target area) and incubated overnight in tilted position. 6 μg of plasmid DNA was used for each transfection. Next day medium was exchanged into medium without supplements and cells were transfected.

Transfection mixes (DNA and PEI) were prepared in 300 μl of DMEM medium without supplements, mixed by inversion and incubated for 15 min at room temperature. After the transfection, cells were incubated for 4 h in 3 ml of DMEM medium without supplements in tilted position, then 6 ml of complete growth DMEM medium was added, which was followed by 24 h incubation of the plate placed at horizontal position (incubation at 27 °C and 5% CO_2_).

## ***Detection of MBA binding to cell surface by immunofluorescence staining***

HEK293T cells transiently transfected with human PD-1/pcDNA6 –myc his (version A) were kept in culture at 37 °C with 5% of CO_2_. Immunofluorescence staining was performed two days after the transfection. SUP-T1 cells were seeded on poly-d-lysine (Gibco) coated 24-well plates. Dishes were coated with 100 μL of 100 μg/ml of poly-D-lysine, incubated for 1 h at 37 °C, then washed three times with PBS and left to dry for ca. 20 min. SUP-T1 cells (0.25 x 10^6^ cells in 0.5 ml of RPMI complete growth medium) were seeded and incubated for 24 h in 37 °C with 5% of CO_2_. Then the immunofluorescence staining was performed.

For HEK293T and SUP-T1 cells, culture medium was discarded from each well, cells were washed once with PBS and then MBA variants in concentration 20 μg/ml (diluted in DMEM medium) were added. Cells treated with MBA variants were incubated for 1 h at 37 °C with 5% of CO_2._ All binding proteins used for staining were dialyzed over the night before staining. After incubation and careful removal of the medium, each well was washed three times with PBS. Mixes containing antibodies were prepared in 200 μl/well of 1.5% BSA-PBS. Antibodies in the staining mix were mouse monoclonal anti-V5-tag–Alexa Fluor 488 conjugated antibody for detection of V5-tag present on the MBA variants and human polyclonal anti-PD-1 antibody for the detection of PD-1 expressed on the surface of transfected cells. Plate was incubated with the antibodies for 1h at room temperature and kept in dark. After incubation, wells were washed with PBS three times and then secondary antibody mix for (200 μl/well of 1.5% BSA-PBS) was applied into the wells and plate was incubated for 1 h at room temperature in dark. Cells were then washed again three times with PBS. For performing imaging using the fluorescence microscope, all wells containing cells were filled with 500 μl of PBS. Processing of the images was done using ImageJ software (Version 1.54f 29). The same procedure was used for a competition staining of MBA variants in the presence of human PD-L1 as a competitor.

## ***Binding kinetics of MBA proteins measured with Ligand Tracer and competition assay***

One day after the transfection of HEK293T cells with human PD-1, culture medium was removed from the cell dish containing cells and 3 ml of fresh DMEM medium was added. Before the measurements using LigandTracer Green Line (Ridgeview Instruments AB, Uppsala, Sweden) with blue-green (488–535 nm) detector, cell dish was inserted in a correct position with the cells oriented downwards (T-target position). The fluorescence signal was expected to be detectable in the green excitation-emission range, as the anti-V5 tag antibody was conjugated with Alexa Fluor 488 (ThermoFisher Scientific). The baseline measurement was done in the absence of the MBA variants and antibodies and lasted at least 15 min or until the fluorescence signal stabilized. All measurements were collected for 15 s with 3 s delay intervals. Association phase measurement was started after 30 min. of incubation of MBA variants with the antibody (incubation in DMEM medium, at room temperature in the dark), when mixes were added to the cell culture media. Measurements were collected for at least 30 min or until the signal was no longer increasing (saturation state). After reaching saturation, higher concentrations of MBA variants were applied. Measurements of the dissociation phase were collected in the absence of antibody and MBA variants (DMEM medium alone) for at least 30 min or when the signal rapidly decreased, indicating dissociation of the protein from the cell surface. Evaluation of the binding was done using TraceDrawer 1.7.1 software. ‘One-to-one’ or ‘One-to-one depletion corrected’ evaluation methods were used for estimation of the MBA variant's kinetic parameters (ka, kd, KD). The same procedure was used for a competition assay of MBA variants in the presence of human PD-L1 as a competitor.

## ***Competition ELISA***

Competition ELISA assays were performed on MAXISORP Nunc 96-well plates. For each assay plate was coated with 1 μg/ml of PD-1 protein (R&D Systems) and incubated in coating buffer for one hour at room temperature. Plate was washed with PBST and blocked overnight with 1% BSA in PBST at 4 °C. MBA variants were prepared in series of dilutions and applied on a plate together with constant 15 nM concentration of PD-L1-Fc protein (RnD Systems). Plate was incubated for 1h at room temperature and then washed with PBST. Anti-PD-L1 antibody was used as a primary antibody and rabbit anti-goat antibody conjugated with HRP was used as a secondary antibody for absorbance signal detection. Absorbance readouts were done at 450 nm.

## ***Determination of K_d_ by micro-scale thermophoresis (MST)***

Determination of the equilibrium dissociation constant (K_d_) of chosen binders was performed using the Monolith NT.115 instrument (NanoTemper Technologies, Germany) by the method of micro-scale thermophoresis. First, the MBA variant dialysed in PBS buffer at 200nM concentration was labeled with His-Tag Labeling Kit RED-tris-NTA 2nd Generation (NanoTemper Technologies, Germany) according to product protocol for 30min at room temperature in the dark. Human PD-1 protein was serially diluted from 4.7 µM to 0.14 nM or from 11.5 µM to 0.35 nM for MBA 197 or MBA414 measurements, respectively. Mixtures of labeled binder and diluted PD-1 protein were loaded into the standard MST capillaries. Experiments were carried out by using 40% of both LED and MST power for MBA197 measurement and 20% LED power and 40% MST power for MBA 414 measurement. For data analysis and graph preparation the Palmist and Gussi softwares were used [1, 2].

# **SI Results**

**
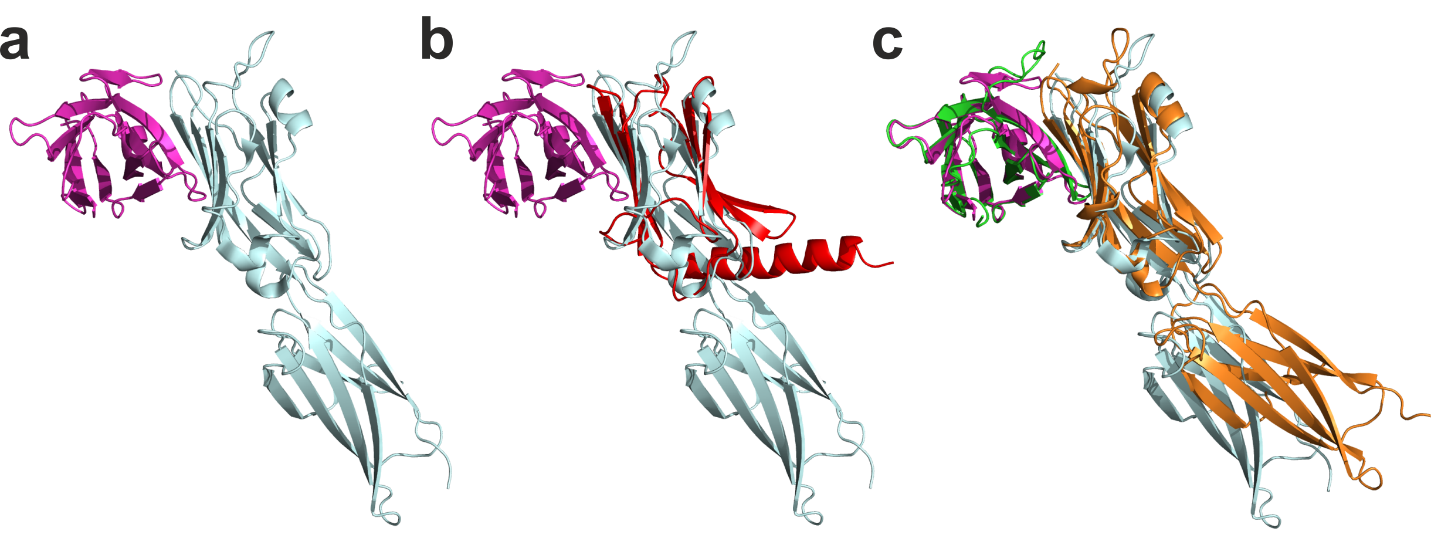
**

**Figur****e S1. Summary of experimental and predicted geometries of PD-1/PD-L1 complexes**. a PD-1/PD-L1 mouse/human complex structure as available from the 3bik PDB structure, the mPD-1 is shown in magenta and the hPD-L1 in light blue cartoon, b superposition of Myomedin scaffold (red cartoon) over the PD-L1 domain interacting with the PD-1 (in light blue cartoon), c Comparison of AlphaFold prediction of the human/human PD-1/PD-L1 complex (green and yellow cartoon) to the 3bik structure of the mPD-1/hPD-L1 complex (magenta and light blue cartoon).

**Table S1:** List of primers used for assembly of myomedin beta sheet combinatorial library where XXX indicates randomized position.

| **Primers** | **Sequence** |
| --- | --- |
| MyoBS_centr _F | GAAGGTCCGAAATACAAAATGCA |
| MyoBS_centr _R | GCCTTCATCTTCATCCTGC |
| MYOM-BS_1F | GAGCGGTAATGCCAAAGTGXXXTATXXXTTCAACXXXAAAGAAATCTTCGAAGGTCCGAAATACAAAATGCA |
| MYOM-BS_2R | TTGAACACGTCACCAACCAGXXXAACXXXTGAXXXATTXXXTGCXXXACCGTCXXXCAGXXXAAAXXXATAXXXGCCTTCATCTTCATCCTGC |
| B-for | AAAAGCGAGCTGGCCGTGGAAATTCTGGAAAAAGGTCAGGTTCGTTTTTGGATGCAGGCAGAAAAACTGAGCGGTAATGCCAAAGTG |
| B-rev | ACCCTGTTTACGAATCCATTCTTGGCGCTGAAATTCTGCTTCTTTTTGCAGTTTTTTGAACACGTCACCAACCAG |

**
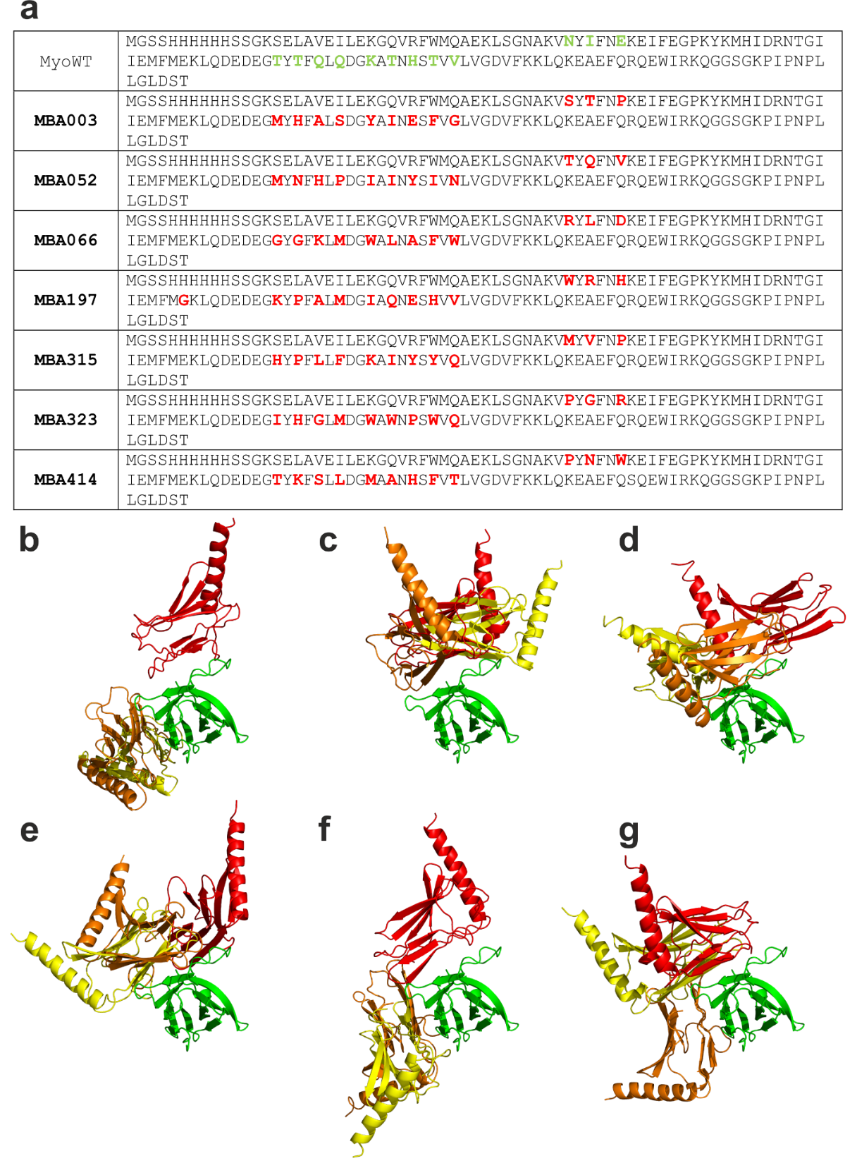
**

**Fig. S2. List of sequences of selected MBA variants and summary of most probable predicted PD-1 binding modes.** **a** Positions of randomized residues of parental un-mutated Myomedin protein are highlighted in green and for particular Myomedin variants in red. **b-g** The model of human PD-1 is shown as green cartoon. Three MBA positions are displayed for each variant with red, orange, and yellow colors in decreasing order of binding mode probability. Panel (**b**) summarizes results for MBA197, (**c**) for MBA414, (**d**) for MBA066, (**e**) for MBA003, (**f**) for MBA315, and (**g**) for MBA323. The source PyMOL session is available from the zenodo repository ( <https://doi.org/10.5281/zenodo.8182102> ).

**Table S2**. Criteria for selection of MBA variants for experiments performed on (**A**) tissue sections and *in vivo experiments* performed on (**B**) mice.

|  | **Criterion** | **Methods** | **Variants selected based on the experimental outcome** |
| --- | --- | --- | --- |
| **A.** Targeting human PD-1 (hPD-1) | Specificity of binding MBA variants to hPD-1 | ELISA – binding to recombinant hPD-1 | MBA003, MBA038, MBA052, MBA066, MBA197, MBA315, MBA323, MBA414 |
|  |  | Immunofluorescence staining of hPD-1-HEK293T cells | MBA003, MBA052, MBA066, MBA197, MBA315, MBA323, MBA414 |
|  |  | Immunofluorescence staining of SUP-T1 and MOLT-4 cells | MBA003, MBA066, MBA197, MBA315, MBA323, MBA414 |
|  | Binding affinity of MBA variants to hPD-1 | Ligand Tracer – using hPD-1-HEK293T cells | MBA003, MBA066, MBA197, MBA315, MBA323, MBA414 |
|  |  | Micro-scale thermophoresis | MBA066, MBA197, MBA414 |
|  | Inhibition potential of MBA variants (competition with hPD-L1) | Competition ELISA – binding to hPD-1 in competition with hPD-L1 | MBA003, MBA323 – inhibiting  MBA066, MBA197, MBA315, MBA414 – not inhibiting |
|  |  | Ligand tracer – using hPD-1-HEK293T cells, experiment performed in presence or absence of hPD-L1 |  |
|  |  | Immunofluorescence staining of hPD-1-HEK293T cells, experiment performed in presence or absence of hPD-L1 |  |
| **B.** Targeting mouse PD-1 (mPD-1) | Purity and stability of ^68^Ga-labeled MBA in human serum | Instant thin layer chromatography | MBA066, MBA197, MBA414 |
|  | Specificity of binding MBA variants to mPD-1 | Immunofluorescence staining of mPD-1-HEK293T cells | MBA066, MBA197, MBA414 |
|  | Binding affinity of MBA variants to mPD-1 | Ligand Tracer – using mPD-1-HEK293T cells | MBA066, MBA197, MBA414 |
|  | Inhibition potential of MBA variants (competition with mPD-L1) | Immunofluorescence staining of mPD-1-HEK293T cells, experiment performed in presence or absence of mPD-L1 | MBA066, MBA197, MBA414 – not inhibiting |

**Table S3.** Estimation of kinetic parameters for six MBA variants binding to human PD-1 cDNA-transfected HEK293T cells analyzed by LigandTracer Green. The evaluation was performed using TraceDrawer software.

| **Variant** | **KD (nM)** | **ka (1/(M*s))** | **kd (1/s)** |
| --- | --- | --- | --- |
| **MBA003** | 25.10 | 6.80 x 10^2^ | 1.71 x 10^-5^ |
| **MBA066** | 6.92 | 6.69 x 10^3^ | 4.63 x 10^-5^ |
| **MBA197** | 29.70 | 2.46 x 10^3^ | 7.30 x 10^-5^ |
| **MBA315** | 17.60 | 1.51 x 10^4^ | 2.65 x 10^-4^ |
| **MBA323** | 6.59 | 3.78 x 10^4^ | 2.49 x 10^-4^ |
| **MBA414** | 8.63 | 2.47 x 10^4^ | 2.13 x 10^-4^ |

**Table S4. Sequence comparison between extracellular part of human and mouse PD-1.** Differences between sequences are marked in red, regions involved in the interaction with PD-L1 are in grey boxes. For the analysis, protein sequences of UniProt entries Q15116 and Q02242.

| Human PD-1  (entry:Q15116) | M**QIP**Q**A**PW**PVV**WAVLQL**G**W**RP**GW**F**L**DS**P**DR**PW**NPP**TF**S**PA**L**L**V**V**T**EG**D**NATFTCS**F**SN**T**SE**SFV**LNW**Y**R**M**SPSNQT**D**K**L**AAF**PEDR**SQP**G**QD**C**RF**RVT**QLPN**GR**DFHM**SVVRA**RRNDSG**T**YLCGAISL**A**PKA**Q**I**K**ES**LR**AEL**R**VTER**RA**E**VP**T**AH**PSPSP**R**P**A**G**Q**FQ**TL**V |
| --- | --- |
| Mouse PD-1  (entry:Q02242) | M**WVR**Q**V**PW**SFT**WAVLQL**S**W**QS**GW**L**L**EV**P**NG**PW**RSL**TF**Y**PA**W**L**T**V**S**EG**A**NATFTCS**L**SN**W**SE**DLM**LNW**N**R**L**SPSNQT**E**K**Q**AAF**CNGL**SQP**V**QD**A**RF**QII**QLPN**RH**DFHM**NILDT**RRNDSG**I**YLCGAISL**H**PKA**K**I**E**ES**PG**AEL**V**VTER**IL**E**TS**T**RY**PSPSP**K**P**E**G**R**FQ**GM**V |

**Table S5.** Estimation of kinetic parameters for three MBA variants binding to murine PD-1 cDNA-transfected HEK293T cells analyzed by LigandTracer Green. The evaluation was performed using TraceDrawer software.

| **Variant** | **KD (nM)** | **ka (1/(M*s))** | **kd (1/s)** |
| --- | --- | --- | --- |
| **MBA066** | 40.5 | 4.77 x 10^2^ | 1.93 x 10^-5^ |
| **MBA197** | 21.4 | 6.32 x 10^3^ | 1.35 x 10^-4^ |
| **MBA414** | 2.48 | 8.31 x 10^3^ | 2.06 x 10^-5^ |

**Table S6**. Radiochemical purity of ^68^Ga-binders and in vitro stability in human serum.

| **^68^Ga-Myomedins** | **Mobile**  **phase** | **Radiochemical purity**  **[%]**  **(*n = 3*)** | **Mobile**  **phase** | ***In* *vitro* stability**  **in human serum [%]** | | |
| --- | --- | --- | --- | --- | --- | --- |
|  |  |  |  | 30 min | 60 min | 120 min |
| ^68^Ga-MyoWT | A | 96.0 ± 0.5 | A | 93.3 | 93.1 | 98.0 |
|  | B | 95.9 ± 0.9 | B | 86.4 | 90.5 | 95.9 |
| ^68^Ga-MBA066 | A | 98.8 ± 0.7 | A | 90.0 | 95.3 | 89.0 |
|  | B | 99.3 ± 0.6 | B | 93.5 | 88.3 | 92.4 |
| ^68^Ga-MBA197 | A | 95.7 ± 1.0 | A | 90.8 | 91.1 | 97.0 |
|  | B | 94.7 ± 0.9 | B | 88.4 | 90.0 | 95.6 |
| ^68^Ga-MBA414 | A | 95.7 ± 2.5 | A | 97.2 | 97.5 | 98.2 |
|  | B | 96.2 ± 1.0 | B | 92.5 | 91.7 | 94.6 |


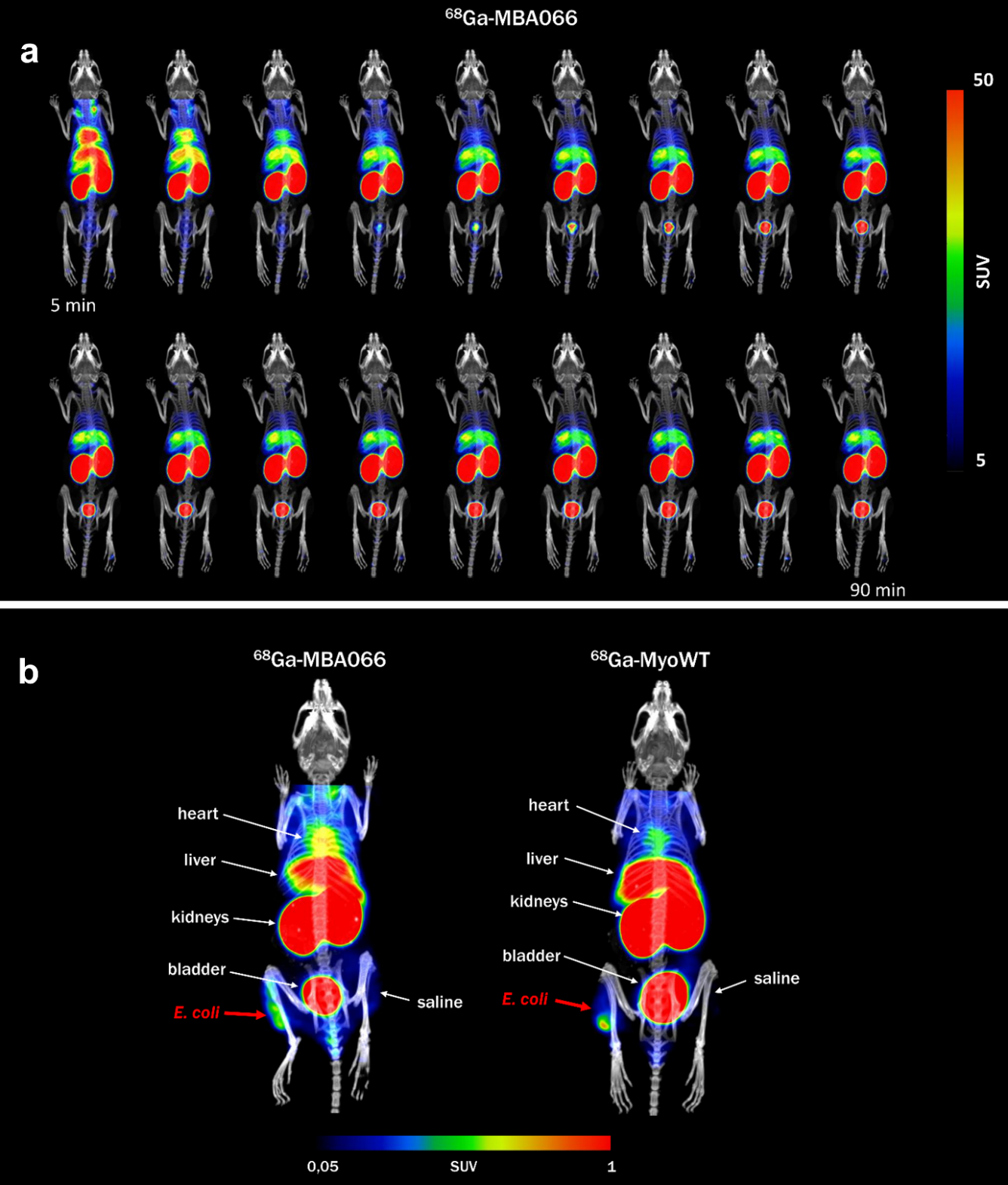


**Fig. S3.** **Distribution of ^68^Galium-labeled MBA066 and MyoWT proteins in *E. coli* infected Balb/c mice.** Static PET/CT imaging. Mice were locally infected by intramuscular injection of *E. coli* cell suspension (5×10^7^ CFU) into the left hind leg, and saline (as control) was injected into the right hind leg. Then, 5 hours after the infection, mice were retro-orbitally injected with ^68^Ga-MyoWT control protein or ^68^Ga-MBA066 variant and scanned 45 minutes later using a PET/CT scanner.


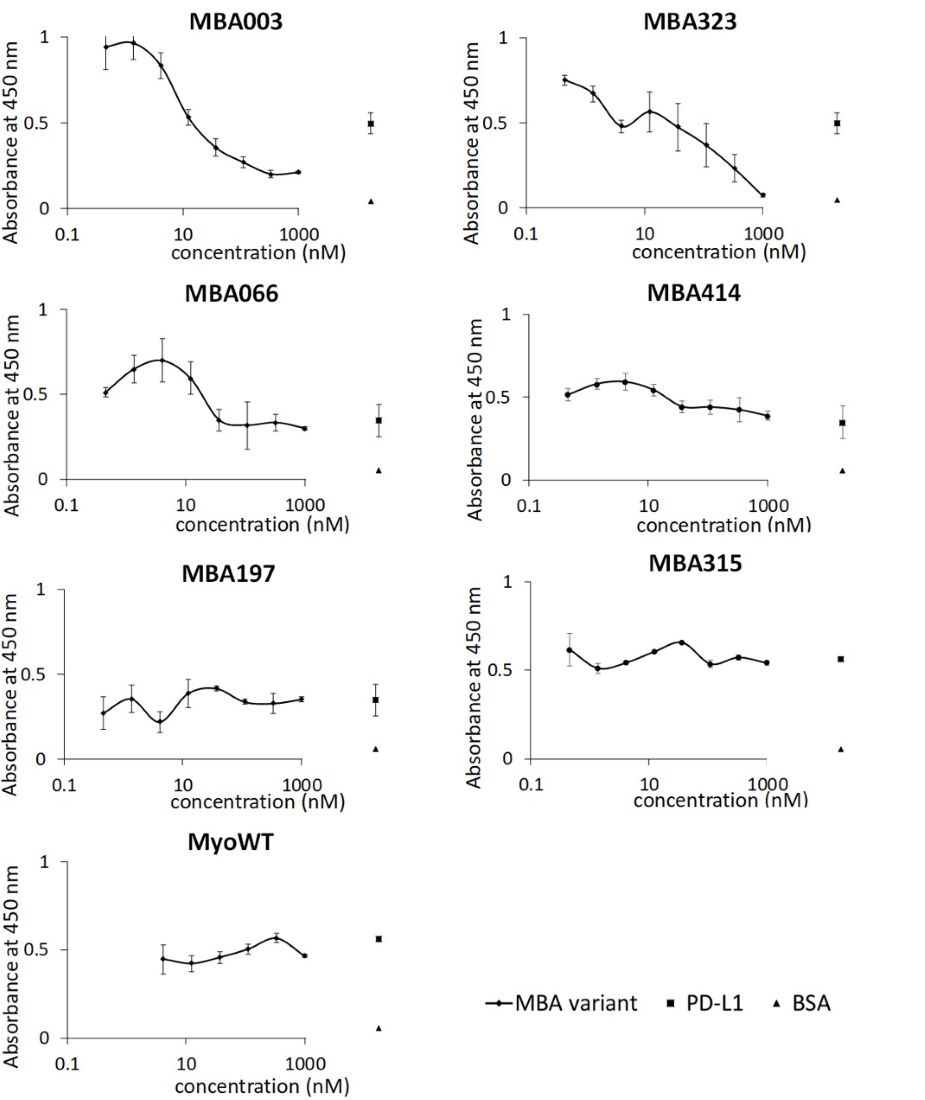


**Fig. S4. Competition of MBA proteins with human PD-L1 for binding to PD-1 using ELISA.** Serially diluted MBA variants were used to compete with 15 nM human PD-L1. Detection of PD-L1 was done using goat anti-human PD-L1 and secondary anti-goat IgG-HRP conjugate. Measuring was performed in triplicates. The rectangle point represents the value for PD-L1 binding in the absence of Myomedins. The triangle point shows the level of signal for binding to bovine serum albumin (BSA).


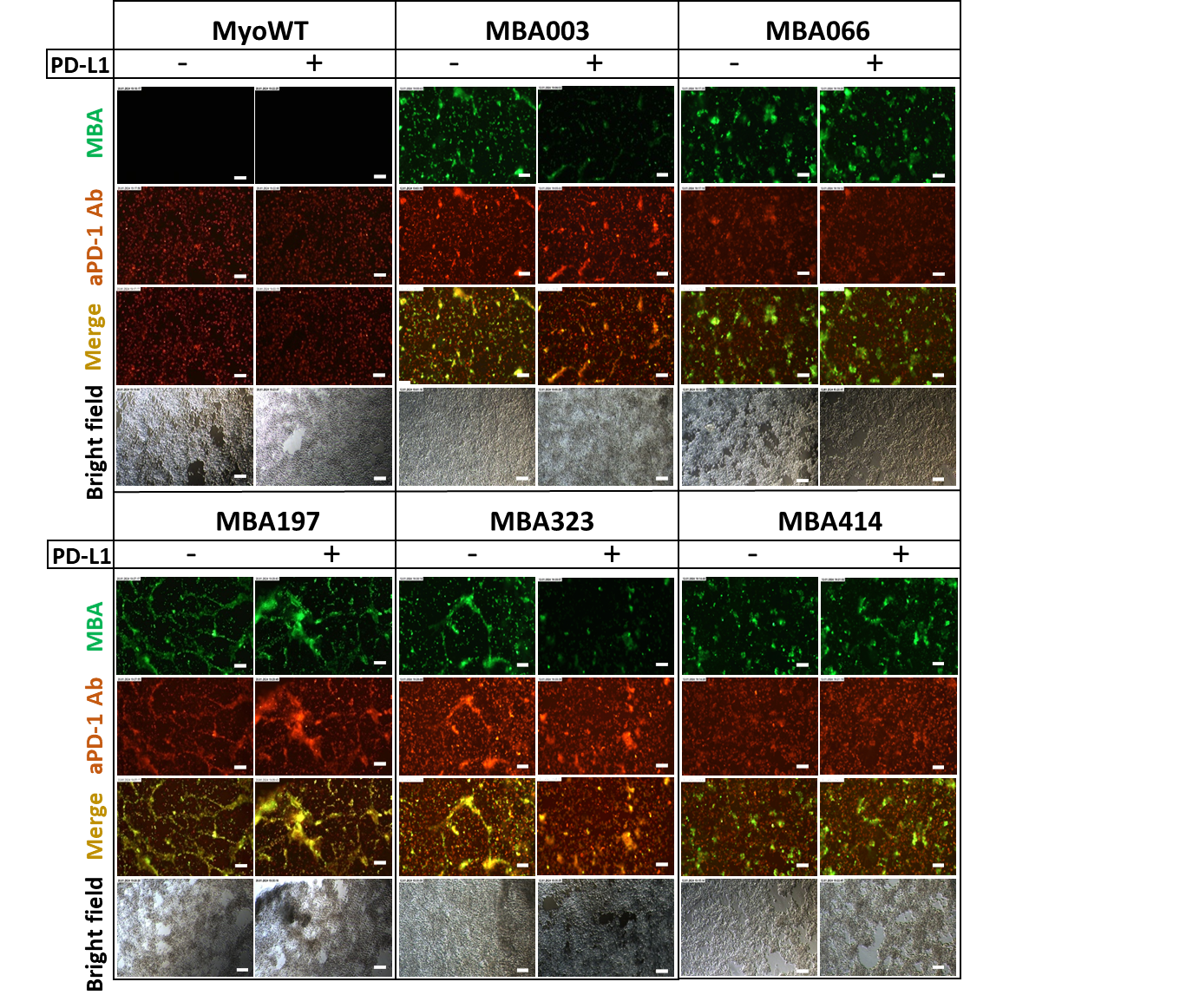


# **Fig. S5. Competition of MBA proteins with human PD-L1 for binding to PD-1-transfected HEK293T cells.** HEK293T cells transiently expressing hPD-1 were treated with 50 nM of PD-L1, then incubated with 5 selected MBA variants and MyoWT as a control (with concentration of 40 μg/ml for each of them). Images present staining for detection of hPD-1 and MBA variants after treatment with PD-L1 (indicated as +) or without treatment with PD-L1 (indicated as -).


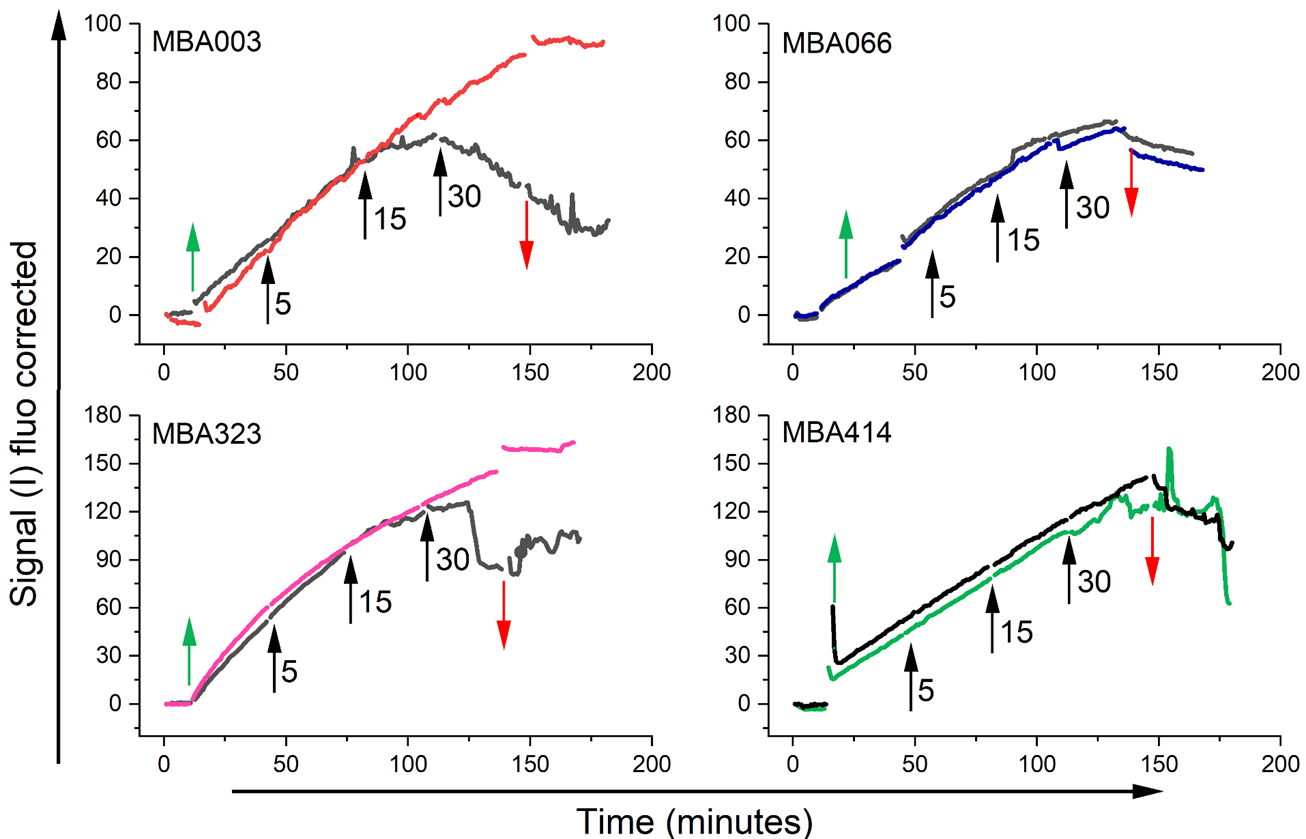


# **Fig. S6. Competition of MBA proteins with hPD-L1 for binding to hPD-1 using LigandTracer.** Binding affinity of MBA003, MBA066, MBA323, and MBA414 to hPD-1 expressed on HEK293T transfected cells in the presence or absence of increasing concentrations of hPD-L1 using LigandTracer Green. After the fluorescence signal stabilized, the association phase was started by addition of 50 nM of the MBA variants to the cell medium (green arrows). During the association phase, three increasing concentrations of hPD-L1 (5, 15 and 30 nM) were added (shown in black arrows). The dissociation phase was started by exchanging the mix of medium containing MBA variant with or without hPD-L1 with the fresh culture medium. Black lines indicate signal from cells which were treated with MBA variant and increasing concentrations of hPD-L1 (5, 15 and 30 nM – indicated by black arrows), whereas lines in distinct colors represent signal from the cells treated with MBA variant in the absence of hPD-L1.

**Table S7.** Comprehensive overview of MBA Myomedin variants used in this study.

| **Experiments *in vitro*** | **Myomedin variant** | | | | | |
| --- | --- | --- | --- | --- | --- | --- |
|  | **MBA003** | **MBA066** | **MBA197** | **MBA315** | **MBA323** | **MBA414** |
| ELISA | Binding to PD-1 | Binding to PD-1 | Binding to PD-1 | Binding to PD-1 | Binding to PD-1 | Binding to PD-1 |
| Micro-scale thermophoresis | N/A^1^ | Kd = 223 nM | Kd = 2.5 nM | N/A | N/A | Kd = 6.9 nM |
| Competition ELISA | Competing | Not competing | Not competing | Not competing | Competing | Not competing |
| IF^2^ staining on hPD-1-HEK293T transfected cells | ++^3^ | + | +++ | ++ | + | ++++ |
| IF staining on SUP-T1 cells | +++ | ++ | ++++ | + | ++ | +++ |
| IF staining on MOLT-4 cells | ++++ | + | ++++ | ++ | ++ | +++ |
| IF staining on mPD-1-HEK293T transfected cells | N/A | + | +++ | N/A | N/A | ++ |
| Ligand Tracer on hPD-1-HEK293T transfected cells | Kd = 25 nM  ka = 6.8 x 10^3^ 1/(m*s)  kd = 1.71 x 10^-5^ (1/s) | Kd = 6.92 nM  ka = 6.93 x 10^3^ 1/(m*s)  kd = 4.63 x 10^-5^ (1/s) | Kd = 29.7 nM  ka = 2.46 x 10^3^ 1/(m*s)  kd = 7.3 x 10^-5^ (1/s) | Kd = 17.6 nM  ka = 1.51 x 10^3^ 1/(m*s)  kd = 2.64 x 10^-4^ (1/s) | Kd = 6.59 nM  ka = 3.78 x 10^4^ 1/(m*s)  kd = 2.49 x 10^-4^ (1/s) | Kd = 8.63 nM  ka = 2.47 x 10^4^ 1/(m*s)  kd = 2.13 x 10^-4^ (1/s) |
| Ligand Tracer on mPD-1-HEK293T transfected cells | N/A | Kd = 40.5 nM  ka = 4.77 x 10^2^ 1/(m*s)  kd = 1.93 x 10^-5^ (1/s) | Kd = 21.4 nM  ka = 6.32 x 10^3^ 1/(m*s)  kd = 1.35 x 10^-4^ (1/s) | N/A | N/A | Kd = 2.48 nM  ka = 8.31 x 10^3^ 1/(m*s)  kd = 2.06 x 10^-5^ (1/s) |
| IF staining on hPD-1-HEK293T transfected cells in presence of hPD-L1 | Slight fluorescence signal decrease upon addition of hPD-L1 | No fluorescence signal decrease upon addition of hPD-L1 – no competition with hPD-L1 | No fluorescence signal decrease upon addition of hPD-L1 – no competition with hPD-L1 | No fluorescence signal decrease upon addition of hPD-L1 – no competition with hPD-L1 | Slight fluorescence signal decrease upon addition of hPD-L1 | No fluorescence signal decrease upon addition of hPD-L1 – no competition with hPD-L1 |
| IF staining on mPD-1-HEK293T transfected cells in presence of mPD-L1 | No fluorescence signal decrease upon addition of mPD-L1 – no competition with mPD-L1 | No fluorescence signal decrease upon addition of mPD-L1 – no competition with mPD-L1 | No fluorescence signal decrease upon addition of mPD-L1 – no competition with mPD-L1 | No fluorescence signal decrease upon addition of mPD-L1 – no competition with mPD-L1 | No fluorescence signal decrease upon addition of mPD-L1 – no competition with mPD-L1 | No fluorescence signal decrease upon addition of mPD-L1 – no competition with mPD-L1 |
| Ligand Tracer on hPD-1-HEK293T transfected cells in presence of hPD-L1 | Decrease in fluorescence signal starting after at 15 nM concentration of added PD-L1 | No decrease in fluorescence signal upon addition of PD-L1 | N/A | N/A | Decrease in fluorescence starting after at 15 nM concentration of added PD-L1 | No decrease in fluorescence signal upon addition of PD-L1 |
| IF staining of PD-1+ T-cells on human tonsil frozen sections | N/A | N/A | N/A | N/A | N/A | High sensitivity staining, pattern overlap with anti-PD-1 antibody |
| Immunostaining of PD-1+ cells on NSCLC tissue | N/A | N/A | N/A | N/A | N/A | High sensitivity staining, pattern overlap with anti-PD-1 antibody |
| Stability of ^68^Ga-labeled MBA in human serum | N/A | After 30 min.: 90.0 (A)^4^ 93.5 (B)  After 60 min.: 95.3 (A) 88.3 (B)  After 120 min.: 89.0 (A) 92.4 (B) | After 30 min.: 90.8 (A) 88.4 (B)  After 60 min.: 91.1 (A) 90.0 (B)  After 120 min.: 97.0 (A) 95.6 (B) | N/A | N/A | After 30 min.: 97.2 (A) 92.5 (B)  After 60 min.: 97.5 (A) 91.7 (B)  After 120 min.: 98.2 (A) 94.6 (B) |
| **Experiments *in vivo*** |  | | | | | |
| *In vivo* imaging of ^68^Ga-labeled MBA/ *ex vivo post mortem* distribution in Balb/c mice | N/A | 90 minutes imaging, analysis of 12 tissues/organs | 90 minutes imaging, analysis of 12 tissues/organs | N/A | N/A | 90 minutes imaging, analysis of 12 tissues/organs |
| Distribution of ^68^Ga-labeled MBA in *E. coli* infected Balb/c mice | N/A | Static PET/CT imaging, intramuscular injection | N/A | N/A | N/A | N/A |

1N/A – not applicable, experiment was not performed, 2IF – immunofluorescence, 3rating of the fluorescence signal intensity for MBA proteins where ++++ signifies the strongest signal and + the weakest, 4A and B indicate two different mobile phases – measured using thin-layer chromatography (A - 0.1 M sodium citrate; B – 1:1 1 M sodium acetate + methanol)


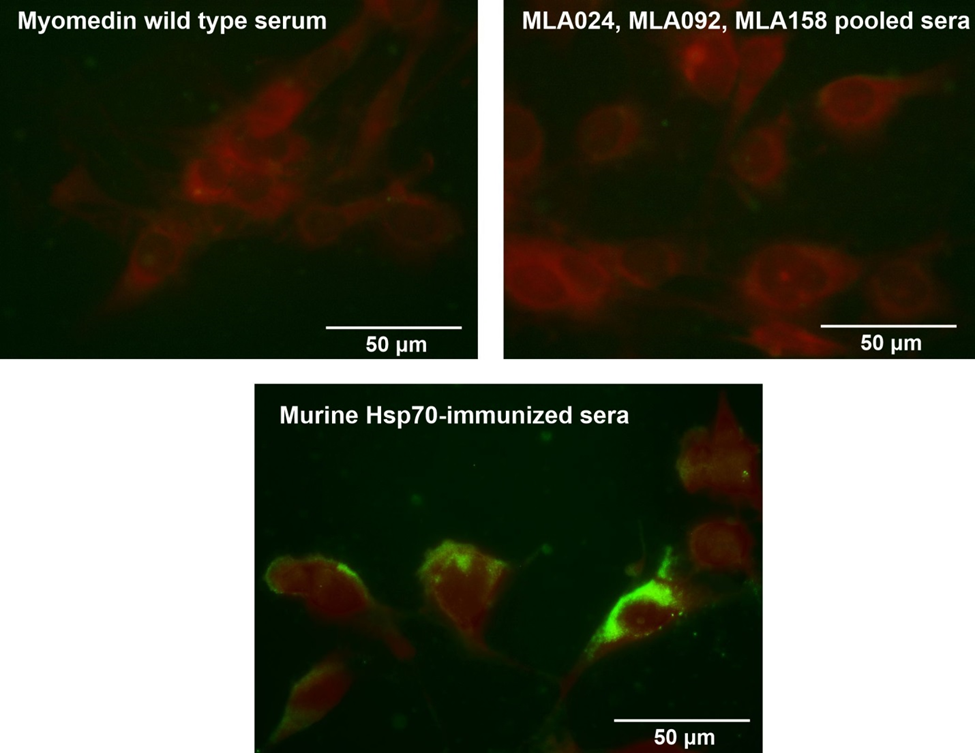


**Fig. S7.** **Myomedins do not elicit autoantibodies after immunization of experimental mice.** To verify whether Myomedins act as potential autoantigens, we incubated pooled Myomedin-immunized sera [3] (dilution 1:50) and naive sera with Triton-X100- (20 min, 0.25% in PBS) permeabilized murine fibroblasts NIH 3T3. After incubation, murine IgG-specific secondary antibody conjugated with Alexa 488 (1:500 in PBS with 0.1 % Tween 20 and 10% fetal bovine serum) was added and cells were stained with Evans blue. We did not confirm detectable reaction of Myomedin-immunized mice sera with cell antigens. As a positive control, we used formerly collected sera from mice immunized with murine heat shock protein 70 kDa (hsp70). In contrast to no reactivity of Myomedin-immunized mice, the sera from hsp70-immunized mice recognized moderately the intracellular antigens, according to observed pattern probably inside mitochondrion or endoplasmic reticulum.

# **SI References**

1. Scheuermann TH, Padrick SB, Gardner KH, Brautigam CA: **On the acquisition and analysis of microscale thermophoresis data**. *Anal Biochem* 2016, **496**:79-93.

2. Brautigam CA: **Calculations and Publication-Quality Illustrations for Analytical Ultracentrifugation Data**. *Method Enzymol* 2015, **562**:109-133.

3. Kuchar M, Kosztyu P, Liskova VD, Cerny J, Petrokova H, Vroblova E, Maly M, Vankova L, Krupka M, Kafkova LR *et al*: **Myomedin scaffold variants targeted to 10E8 HIV-1 broadly neutralizing antibody mimic gp41 epitope and elicit HIV-1 virus-neutralizing sera in mice**. *Virulence* 2021, **12**(1):1271-1287.
